# Novel Polyethers from Screening *Actinoallomurus* spp.

**DOI:** 10.3390/antibiotics7020047

**Published:** 2018-06-14

**Authors:** Marianna Iorio, Arianna Tocchetti, Joao Carlos Santos Cruz, Giancarlo Del Gatto, Cristina Brunati, Sonia Ilaria Maffioli, Margherita Sosio, Stefano Donadio

**Affiliations:** 1 NAICONS Srl, Viale Ortles 22/4, 20139 Milano, Italy; miorio@naicons.com (M.I.); atocchetti@naicons.com (A.T.); giancarlo_delgatto@hotmail.it (G.D.G.); smaffioli@naicons.com (S.I.M.); msosio@naicons.com (M.S.); 2 KtedoGen Srl, Viale Ortles 22/4, 20139 Milano, Italy; maildocruz@gmail.com (J.C.S.C.); cbrunati@naicons.com (C.B.)

**Keywords:** *Actinoallomurus*, antibiotics polyethers, screening

## Abstract

In screening for novel antibiotics, an attractive element of novelty can be represented by screening previously underexplored groups of microorganisms. We report the results of screening 200 strains belonging to the actinobacterial genus *Actinoallomurus* for their production of antibacterial compounds. When grown under just one condition, about half of the strains produced an extract that was able to inhibit growth of *Staphylococcus aureus*. We report here on the metabolites produced by 37 strains. In addition to previously reported aminocoumarins, lantibiotics and aromatic polyketides, we described two novel and structurally unrelated polyethers, designated α-770 and α-823. While we identified only one producer strain of the former polyether, 10 independent *Actinoallomurus* isolates were found to produce α-823, with the same molecule as main congener. Remarkably, production of α-823 was associated with a common lineage within *Actinoallomurus*, which includes *A. fulvus* and *A. amamiensis*. All polyether producers were isolated from soil samples collected in tropical parts of the world.

## 1. Introduction

Antimicrobial resistance among bacterial pathogens is becoming a major threat to human health and well-being. While different approaches can be deployed to mitigate and delay the insurgence and spread of antibiotic resistance, it is also clear that we will need a constant supply of new antibiotics, especially new chemical classes not affected by current resistance mechanisms. However, new chemical classes of antibiotics have been extremely difficult to discover from combinatorial and chemical libraries and microbial products still represent a major source of drug leads as antibiotics [1].

One of the main issues with antibiotic discovery based on microbial products is the probability of rediscovering known metabolites. This requires introducing one or more elements of novelty in the screening with respect to past efforts [2,3]. An attractive element of novelty can be represented by using novel strains, for example a taxonomic group that has not witnessed extensive analyses of its secondary metabolites, since taxonomic diversity can be seen as a surrogate for chemical diversity [4]. The main idea behind this concept is that organisms that have been subjected to different evolutionary pressures have developed unique biology to survive and, for some taxa, secondary metabolites are an important part of their biology. However, since production of secondary metabolites is not distributed equally among all species, it is important to select a taxon with a high potential to produce bioactive compounds in order to increase the probability of finding new compounds with a reasonable screening effort. Following this rationale, we initiated over a decade ago a project aimed at finding taxonomically divergent filamentous *Actinobacteria*, which led to the discovery of several novel taxa, including new suborders, families and genera [5,6,7,8]. One of the new taxa, originally designated as “alpha” [5], turned out to coincide with the genus *Actinoallomurus* (family *Thermomonosporaceae*), formally described in 2009 [9] with new entries added since [10,11,12,13,14,15]. With proper isolation methods, strains belonging to the genus *Actinoallomurus* could be effectively retrieved from a variety of soil samples, enabling the creation of a consistent collection of about 1000 isolates [2,12].

Strains belonging to the genus *Actinoallomurus* have been shown to produce a variety of metabolites [12,15,16,17,18,19] originating from different biosynthetic pathways. In this study, we explored 200 randomly picked *Actinoallomurus* isolates from the NAICONS strain collection. Together with the metabolites previously described [16,17,18,19], we analyzed the antibacterial compounds produced by 37 strains. This set of compounds includes two novel polyethers, as described here.

## 2. Results and Discussion

### 2.1. The Screened Set

The selected *Actinoallomurus* strains were isolated from a variety of samples collected in different continents, and representing diverse environments such as densely vegetated areas, sulfur-enriched craters of volcanic origin, and plant rhizosphere. The geographic distribution of the screened strains is listed in Table 1. 

Three extract types were prepared from the strains―see Material and Methods―and evaluated for their ability to inhibit growth of *Staphylococcus aureus* and of a *ΔtolC* mutant of *Escherichia coli*. Overall, 104 and 17 strains produced at least one extract with activity against *S. aureus* and *E. coli*, respectively. All extracts with activity against *E. coli* were also active against *S. aureus*. The highest activity was observed in the mycelium and the ethyl acetate extracts at comparable frequency (57 and 46, respectively), and only in one case was the ethyl acetate exhaust extract more active. Except perhaps for an under-representation of active strains isolated from Asian samples, there was no apparent effect of the continent of origin on the frequency of anti-staphylococcal activity (Table 1). Positive extracts were analyzed as described under Material and Methods, leading to preliminary information on the chemical identity of the identified compounds. We report below the characterization of the molecules identified from 37 of the active strains.

### 2.2. Coumermycins, Spirotetronates, Lantibiotics and Diketopiperazines

Coumarin antibiotics target bacterial DNA gyrase and one member of this family, novobiocin, has been used to treat bacterial infections in humans caused by Gram-positive bacteria [20]. Coumermycins are other member of this family with higher antibacterial activity than novobiocin [20]. We have previously reported that the *Actinoallomurus* sp. K275, belonging to the Alp18 phylotype, produced several members of the coumermycin complex [12]. In the course of screening the 200 strains, two additional coumermycin producers were identified: strains ID145250 and ID145519, belonging to the Alp22 phylotype. The main coumermycin congeners produced by these three strains were A2, D1 and A1, respectively. Relevant data are shown in Supplementary Materials Figure S1. Coumermycins have been reported mostly from *Streptomyces* spp. [20].

Tetronate-containing polyketide natural products represent a large and diversified family of microbial metabolites with different bioactivities [21]. Halogenated spirotetronates designated NAI-414 A and B were previously described as the main products of *Actinoallomurus* sp. ID145414 [16]. During our screening, two strains (ID145260 and ID145814) were found to produce a molecule with *m/z* [M−H]^−^ 839, an isotopic pattern compatible with the presence of two chlorines and a UV spectrum with maxima at 228 and 268 nm (see Supplementary Materials Figure S2). These properties closely resemble those reported for NAI-414 and actually match those reported for pyrrolosporin, a compound structurally related to NAI-414 but containing an additional unsaturation in the polyketide backbone. Pyrrolosporin was previously reported as a metabolite from a *Micromonospora* sp. [22,23].

Ribosomally synthesized and post-translationally modified peptides represent a rapidly expanding family of microbial metabolites, with lantibiotics as one of better known representatives [24]. *Actinoallomurus* sp. ID145699 was previously reported to produce the chlorinated lantibiotic NAI-107 and its brominated variant in Br-supplemented medium [19]. In the course of our screening, we identified strain ID145640 as an additional NAI-107 producer, with the known variations at Trp4 (hydrogen or chlorine) but just zero or one hydroxylation at Pro14 (Supplementary Materials Figures S3 and S4). Previously, NAI-107 was reported as the product of two independent *Microbispora* isolates [25,26].

**Figure 1:**
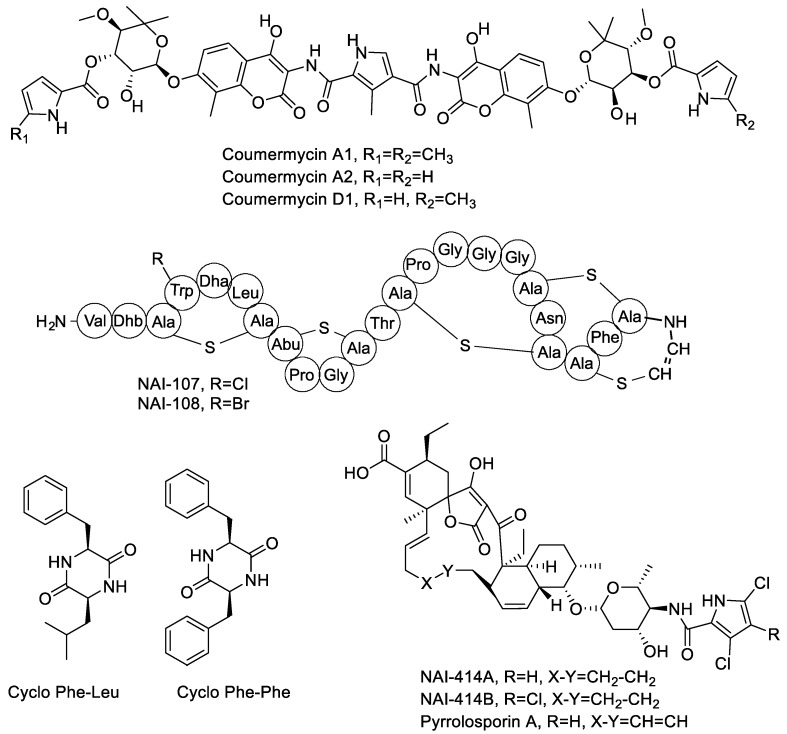
Chemical structures of molecules produced by *Actinoallomurus* and described in Section 2.2.

Diketopiperazines represent a broad family of cyclized dipeptides produced by a large variety of microorganisms [27]. It is thus no surprise that one of the strains in the present work, *Actinoallomurus* sp. ID145219, produced two compounds with activity against *S. aureus* that were identified as cyclo-Phe-Leu and cyclo-Phe-Phe (see Supplementary Materials Figure S5). The structures of the metabolites mentioned in Section 2.2 are illustrated in Figure 1.

### 2.3. Aromatic Polyketides

During the course of our screening, we frequently encountered strains producing aromatic polyketides. Identified products included the allocyclinones, hyper-halogenated angucyclinones detected from twelve independent strains belonging to three different phylotypes [17]; the paramagnetoquinones, highly paramagnetic tetracenes produced by three independent strains belonging to three different phylotypes [18]; three producers of the related dihydrobenzo-(alpha)-naphthacenequinones pradimicin (strains ID145114 and ID145318) and benanomicin (strain ID145226) (see Supplementary Materials Figure S6). Pradimicin and benanomicin present a common polyketide core decorated with a disaccharide unit and differ for the presence/absence of an *N*-methyl on the aminated-sugar. Both compounds were previously reported as products of *Actinomadura* spp., with pradimicin produced by a confirmed species of the genus, *Actinomadura hibisca* [28]. Benanomicin had already been reported as a metabolite of *Actinoallomurus* strain K15 [12].

**Figure 2:**
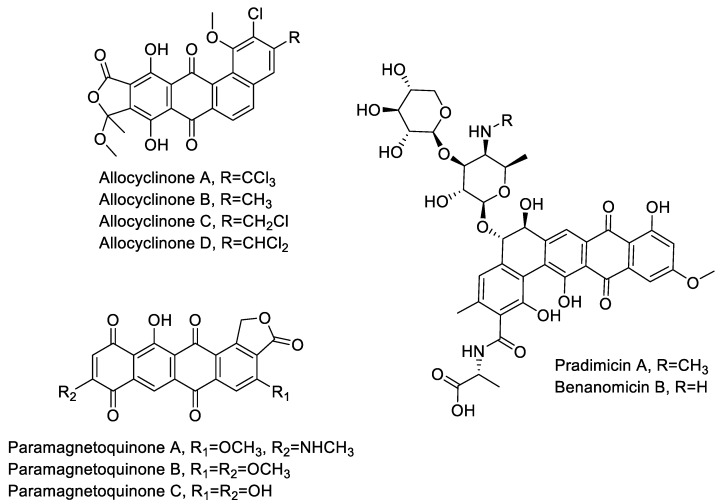
Chemical structures of aromatic polyketides produced by *Actinoallomurus* and described in Section 2.3.

Overall, this brief survey of aromatic polyketides indicates that *Actinoallomurus* spp. are capable of producing decaketides (i.e., paramagnetoquinones), undecaketides (i.e., allocyclinones, presumably undergoing oxidative ring cleavage after polyketide formation) and dodecaketides (i.e., pradimicin and benanomicin). The structures of these metabolites are shown in Figure 2.

### 2.4. Polyethers

The extracts from several strains presented large inhibition halos against *S. aureus* but little or no activity against the *E. coli ΔtolC* strain. Upon resolution by high performance liquid chromatography (HPLC), the active fractions showed a retention time of 5–11 min and, with one exception, had no ultraviolet (UV) absorption. Mass spectrometry (MS) analysis indicated the presence of *m/z* signals consistent with the formation NH_4_^+^ and Na^+^ adducts but with no detectable H^+^ adducts. It should be noted that the extraction procedure and the liquid chromatography (LC)-MS eluent do not contain ammonium or sodium ions. Hence, the observation of NH_4_^+^ and Na^+^ adducts suggests a high cation-binding ability of the active molecules. Moreover, the fragmentation pattern showed losses of 44 amu (free carboxylic acid) and 62 amu (decarboxylation and dehydratation). As demonstrated below, we identified three distinct polyether families within twelve strains: one new compounds, designated α-823, produced by ten independent isolates; an additional new polyether, designated α-770, and the previously reported octacyclomycin, produced by one strain each. Table 2 lists the identified polyether-producing *Actinoallomurus* isolates, along with their origins, accession number of the 16S rRNA gene sequences and the *m/z* value of the most abundant congener. 

**Figure 3:**
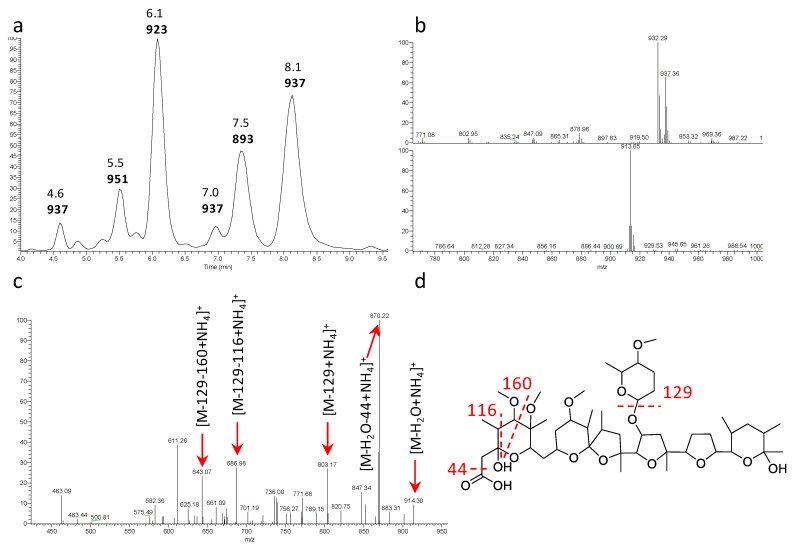
Analysis of α-823. (**a**) Base peak chromatogram of the 4.0–10.0 min portion with retention times and *m/z* [M+MH4]^+^ values. Data obtained with a partially purified extract of *Actinoallomurus* sp. ID145823 (see Table S1 and Figure S7 for the congeners comparison); (**b**) mass spectrometry (MS) at 8.1 min in positive (above) and negative (below) ionization mode; (**c**) MS^2^ analysis of *m/z* [M+NH_4_]^+^ 932; (**d**) putative fragmentation pathway for α-823.

**Figure 4:**
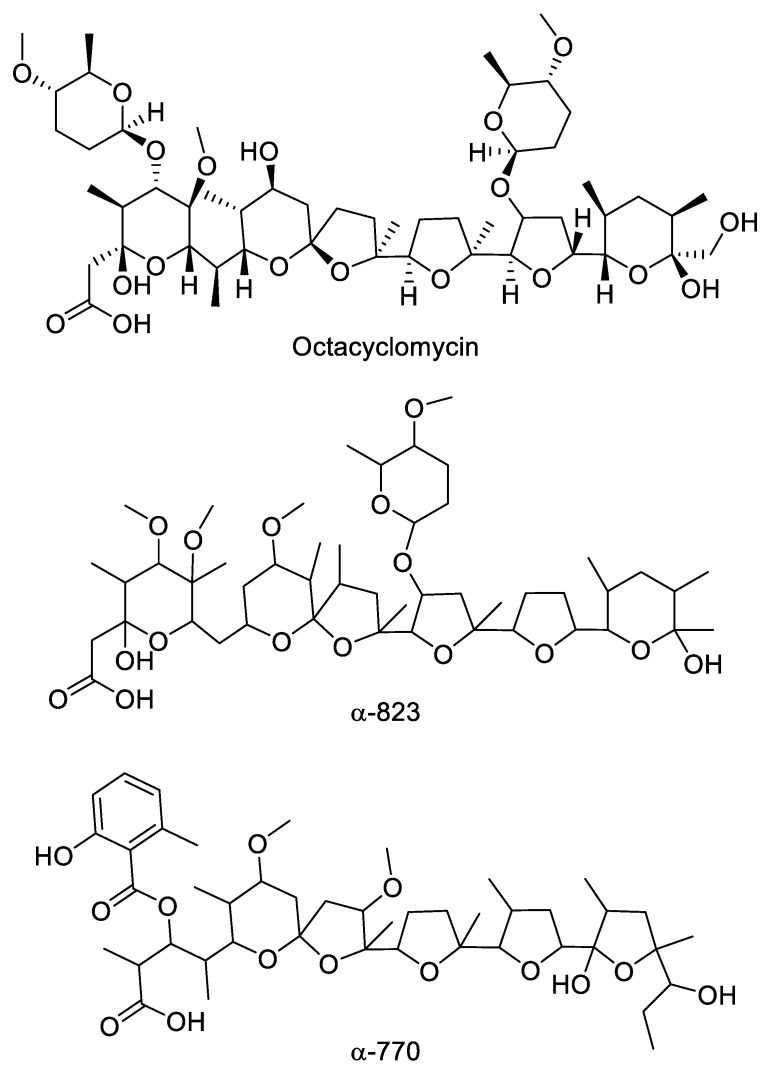
Chemical structure of polyethers produced by *Actinoallomurus* and described in Section 2.4.

Several strains produced a likely polyether with major *m/z* signals [M+NH_4_]^+^ 932 and [M+Na]^+^ 937 (see Figure 4a,b for representative example). The metabolite produced by all these strains appeared identical (Table 2) and those from strain ID145823 were analyzed in detail. The strain produced a complex of related molecules (Figure 4a; Supplementary Materials Table S1) with similar HPLC retention times (they all eluted at ≥90% acetonitrile; see Supplementary Materials Figure S7), appearing as both [M+NH_4_]^+^ and [M+Na]^+^ adducts, and with similar fragmentation patterns (Supplementary Materials Figure S7). The deduced molecular formulae indicate that the congeners varied in methyl group(s) and oxygen(s) (Supplementary Materials Table S1). The structure of the major congener, designated α-823, was elucidated by a combination of NMR (Supplementary Materials Figures S8–S13) and HR-ESI-MS (Figure S14) and MS/MS analyses. The molecular formula was defined as C_48_H_82_O_16_Na (calculated 937.5495 [M+Na]^+^, found 937.5510 [M+Na]^+^). The analysis of ^1^H-monodimentional spectrum revealed the presence of four singlet and six doublet methyl signals, along with four methoxy groups. Moreover, several diastereotopic methylene signals were observed using 2D-HSQC (bi-dimensional Heteronuclear Single Quantum Coherence) experiments, indicating CH_2_ inserted into rigid structures or close to stereocenters. COSY (COrrelated SpectroscopY) and TOCSY (TOtal Correlated SpectroscopY) analyses, along with HMBC (Heteronuclear Multiple Bond Correlation, resulted in the structure shown in Figure 3. α-823 consists of a C_30_ chain with three substituted tetrahydrofuranes and three substituted tetrahydropyranes. Tetrahydrofurane C carries a deoxysugar (Figure 4). Structurally, α-823 closely resembles the polyether SF-2361, produced by an *Actinomadura* sp. [29,30]. Despite an identical molecular formula, α-823 carries a methyl at C-6, while a methyl group in SF-2361 has been assigned to C-2. Indeed, in the α-823 spectrum C-2 is a free methylene with δ_H_ signal at 2.18–2.54 ppm and δ_C_ at 44.7 ppm (due to the proximity to a hemiacetal and a carboxylic acid), while C-6 carries no proton (δ_C_ at 78.6 ppm) and shows HMBC correlations with a methoxy at 3.38 ppm and a singlet methyl at 1.16 ppm.

Strain ID145817 was found to produce a bioactive compound eluting at 9.0 min with no UV adsorption. Upon MS analysis, it showed *m/z* signals [M+NH_4_]^+^ 1034 and [M+Na]^+^ 1039, corresponding to the NH_4_^+^ and Na^+^ adduct of a molecule of 1016 amu, with major fragments at 990–995 and 972–977 (Supplementary Materials Figure S15). Additionally, a Δ*m*/*z* of 129 suggested the elimination of a deoxysugar. All these properties are consistent with the metabolite produced by strain ID145817 being identical to octacyclomycin, a di-glycosylated polyether previously reported from a *Streptomyces* sp. [31]. NMR analysis of the purified compound confirmed this hypothesis showing signals identical to those reported in literature for octacyclomycin (data not shown). The structure of octacyclomycin is reported in Figure 4. Octacyclomycin, SF-2361 and α-823 derive from a C_30_ chain with identical sequence of tetrahydrofuranes and tetrahydropyranes, but differ for the number and position of methyls, oxygens and glycosyl moieties.

**Figure 5:**
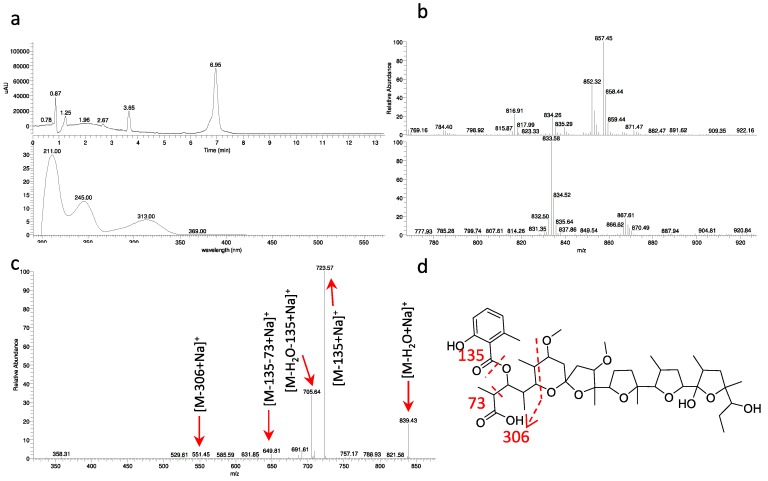
Analysis of α-770. (**a**) UV chromatogram at 230 nm and UV spectrum of 6.9-min peak. Data obtained with a partially purified extract of *Actinoallomurus* sp. ID145770; (**b**) MS at 6.9 min in positive (above) and negative (below) ionization mode; (**c**) MS^2^ of *m/z* [M+NH_4_]^+^ 852; (**d**) putative fragmentation pathway for α-770.

Strain ID145770 was found to produce an active peak eluting at 6.9 min and showing the polyether-diagnostic *m/z* signals [M+NH_4_]^+^ 852 and [M+Na]^+^ 857, corresponding to a molecule of 834 amu (Figure 5a,b). Unlike the other polyethers, however, this peak showed a UV signal with maximum at 314 nm (Figure 5a) and a Δ*m*/*z* 135 upon MS fragmentation, consistent with presence of a methylsalicylate moiety (Figure 5c), a chromophore previously found in the polyether cationomycin [32]. The active molecule was produced along with a related, Δ*m*/*z* +14 species, consistent with the presence of an extra methyl group atom, as listed in Table S3 and shown in Figure S16. The structure of the major congener α-770 was elucidated by a combination of NMR (Figures S17–S22), HR-ESI-MS (Figure S23) and MS/MS analyses (Figure 5). The molecular formula was defined as C_45_H_70_O_14_Na (calculated 857.4658 [M+Na]^+^, found 857.4695 [M+Na]^+^). The analysis of ^1^H- and HSQC spectra revealed the presence of 2 methoxy along with 10 methyl groups, with four of them devoid of multiplicity. Moreover, several diastereotopic methylene signals were observed, indicating CH_2_ inserted into rigid structures or close to stereocenters. The 2D-NMR-experiments allowed assigning the carbons in a structure consisting four substituted tetrahydrofuranes and one substituted tetrahydropyrane, as shown in Figure 4. The overall structure of α-770 is similar to that of cationomycin, produced by an *Actinomadura* sp. [32]. Both polyethers consists of a C_27_ chain with identical positioning of the methyl groups, suggesting they a common origin from incorporation of the same sequence of propionate and acetate precursors [33]; and both polyethers carry a 6-methylsalicylate moiety linked in an ester bond the C-3 hydroxyl. However, despite these similarities and the small mass difference (16 amu), α-770 and cationomycin differ significantly in the hydroxyl and methoxy decorations. Indeed, 2D-HMBC correlations established that α-770 lacks the hydroxyls at positions 15 and 5′, which are present as methoxy groups in cationomycin, as well as the hydroxyl at position 3. In contrast, α-770 carries methoxys at positions 11 and 21, while cationomycin has no hydroxyls at those positions.

The carbon chain of polyethers is assembled by type I PKSs, followed by ring formation by dedicated epoxidases [33]. The carbon chains of α-770 and α-823 are likely to derive from trideca- and pentadeca-ketide precursors, respectively. In addition, the 6-methylsalicylate unit of cationomycin has been show to result from acetate incorporation [34], consistent with the involvement of a type III PKS system.

The antimicrobial activity of polyethers is strictly connected to their ability to insert into cellular membranes and alter transport of metal cations, which leads to changes in the osmotic pressure inside the cytoplasm and cell death [30,35]. However, polyethers generally lack cellular selectivity. The antibacterial activities of α-823 and α-770 are reported in Table 3. They show potent activities against most Gram-positive bacteria, with minimal inhibitory concentrations (MICs) well below 1 µg/mL for α-770, the most active of the three compounds, with 2–4 times lower MICs than salinomycin against most of the tested strains. The polyether α-823 was generally 4–16 times less active than α-770, except for an increased activity against *Mycobacterium smegmatis*. No activities were detected against Gram-negative strains (not shown), except for *Moraxella catarrhalis*.

Some polyethers (e.g., salinomycin, monensin) are commercially used as coccidiostatic agents and salinomycin has also been evaluated as anticancer agents [36]. Recently, salinomycin and other ionophores have been shown to have transmission blocking activity against the etiological agent of malaria [37]. When tested against one chloroquine-sensitive and one chloroquine-resistant strain of *Plasmodium falciparum*, α-770 and α-823 showed inhibitory activity in the 2–10 nM range comparable to those of salinomycin [37]. It should be noted that, although there are over 120 reported polyethers in the literature, their mechanism of action has been studied on a limited number of molecules [30] and we are not aware of studies aimed at making polyethers selective towards a particular cell type.

**Figure 6:**
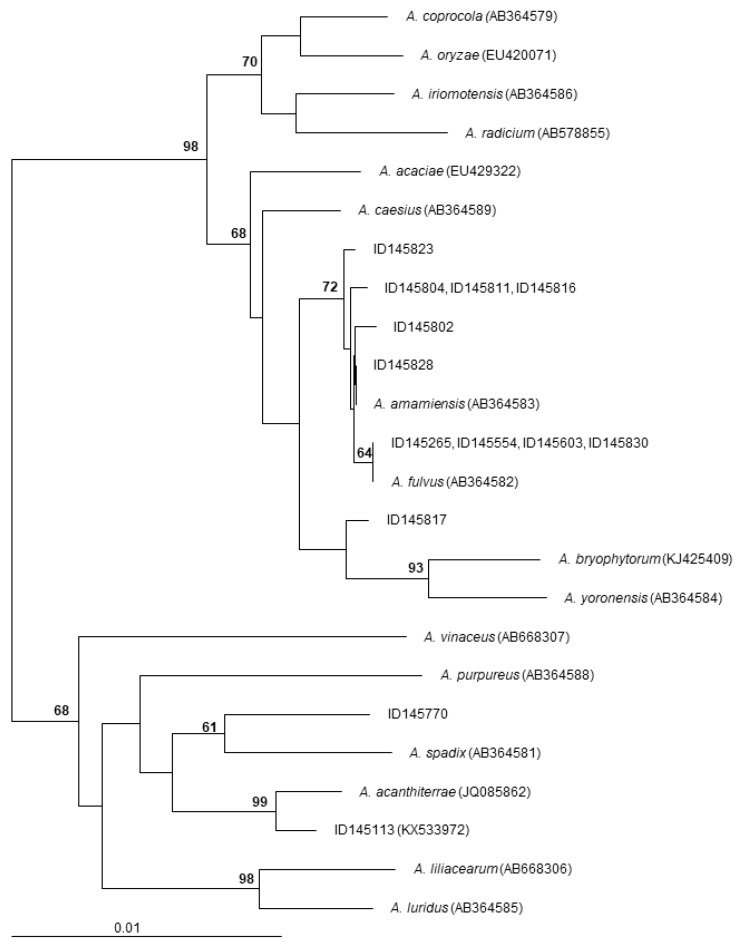
Neighbor-joining tree showing the phylogenetic position of polyether-producing *Actinoallomurus* strains. Type-strains of all described *Actinoallomurus* species are included. The sequence of the paramagnetoquinone producer *Actinoallomurus* sp. ID145113 is also included. The tree is based on 1309 unambiguously aligned positions in the 16S rRNA gene sequences. Numbers at the nodes are bootstrap values based on 100 resamplings; only values higher than 60 are shown. Scale bar represents 1 inferred substitutions per 100 nucleotides. The tree was rooted using *Streptosporangium roseum* 16S rRNA gene sequence (X89947) as outgroup.

The 16S rRNA gene sequences of the polyether-producing *Actinoallomurus* strains of Table 2 were determined and compared to those of all described *Actinoallomurus* species. The results are shown in Figure 6. All the ten α-823 producers cluster together in a compact branch that includes the type strains *Actinoallomurus fulvus* and *A. amamiensis*: specifically, the identical 16S rRNA gene sequences from strains ID145265, -145554, -145603 and -145830 are 100% identical to that from *A. fulvus*; the 16S rRNA genes sequences from ID145802 and -145828 are 99.9% and 100% identical, respectively, to that from *A. amamiensis*; while the identical 16S rRNA gene sequences from strains ID145804, -145811 and -145816 and that from strain ID145823 are less related to those of described species (Figure 6). The octacyclomycin producer ID145817 is less closely related (≤99.1% identity) to *A. bryophytorum* and *A. yoronensis*, although all these strains belong to a related phylogenetic branch. The α-770 producer, instead, is distantly related to *A. spadix* (98.7% identity) and belongs to an unrelated branch that includes, among others, strain ID145113, the producer of the aromatic polyketide paramagnetoquinone [18]. Remarkably, all polyether-producing *Actinoallomurus* strains were isolated from soil samples of tropical origin (Table 2), notwithstanding that 64% of the screened strains were of non-tropical origin (mostly from Europe; see Table 1). These observations suggest that the branch including *A. fulvus*, *A. amamiensis* and the α-823 producers might consist of cosmopolitan strains and that polyether production might be mostly associated with *Actinoallomurus* strains from tropical environment. Previous studies have established a correlation between different classes of secondary metabolites and geographic origin [38,39], although we are unaware of previous reports on biogeography of polyether production.

## 3. Materials and Methods

### 3.1. Bacterial Strains and Media

*Actinoallomurus* strains are from the NAICONS strain library. Each strain was propagated on S1-5.5 plates (60 g/L oatmeal, 18 g/L agar, 1 mL/L Trace Elements Solution) at 30 °C for two to three weeks. From these plates, the grown mycelium was used to inoculate AF-A medium (10 g/L dextrose monohydrate, 4 g/L soybean meal, 1 g/L yeast extract, 0.5 g/L NaCl, 1.5 g/L 2-(*N*-morpholino) ethanesulfonic acid, pH adjusted to 5.6) in shake-flasks. After 8 days in a rotatory shaker (200 rpm) at 30 °C, cultures were harvested and extracted (see Section 3.2). 

PCR amplifications with the eubacterial primers F27 and R1492 and phylogenetic analyses of the 16S rRNA gene sequences were performed as previously described [40]. The 16S rRNA gene sequences have been deposited in GenBank, as listed in Table 2.

### 3.2. Preparation of Extracts

Three different extracts were prepared from each culture. The culture was centrifuged at 16,000 rcf for 5 min and the resulting pellet was resuspended in 0.4 vol ethanol, while the supernatant was used for ethyl acetate extraction (see below). After shaking 1 h at 55 °C, the suspension was centrifuged once more (16,000 rcf, 5 min) and the supernatant transferred to a new tube, dried under vacuum and resuspended in 10% DMSO at 0.2× the original culture volume. This extract was designated as the mycelium extract. 

The supernatant from the first centrifugation step above was extracted with 0.5 vol ethyl acetate. After mixing and phase separation, the organic phase was transferred to a new tube and the aqueous phase extracted again with further 0.5 vol ethyl acetate. The two organic phases were combined, dried and resuspended at 5× the original concentration in 10% DMSO. This extract was designated as EtAc extract. The exhausted aqueous phase was also retained and tested as such. 

### 3.3. Antibacterial Assays

The screening was performed by agar diffusion, using plates containing 15 mL of Müller-Hinton Agar and inoculated with 5 × 10^5^ CFU/mL of the indicator strain. Strains used in this assay were *Staphylococcus aureus* ATCC 6538P and *Escherichia coli* L4242, a *ΔtolC* derivative of MG1061. After spotting 20 μL of the resuspended extract, plates were incubated 18–20 h at 37 °C. After HPLC fractionation, bioactive fractions were identified using the same methodology.

MIC determinations of purified compounds were performed by broth micro dilution in sterile 96-well polystyrene microtiter plates according to CLSI guidelines, using Müller Hinton broth (Difco Laboratories) containing 20 mg/L CaCl_2_ and 10 mg/L MgCl_2_ for all strains except for *Streptococcus* spp., which were grown in Todd Hewitt broth. Strains were inoculated at 5 × 10^5^ CFU/mL and incubated at 37 °C for 20−24 h. Strain with an L prefix are from the NAICONS pathogens library.

### 3.4. Analytical Procedures 

For monitoring metabolites production analytical HPLC was performed on Shimadzu Series 10 spectrophotometer (Kyoto, Japan), equipped with a reverse-phase column, LiChrospher RP-18, 5 μm, 4.6 × 125 mm (Merck, Darmstadt, Germany). Phase A was 0.1% trifluoroacetic acid (TFA), phase B acetonitrile, and the flow rate was 1 mL/min. Resolution was achieved with a linear gradient from 10% to 36% phase B in 5 min; from 36% to 50% phase B in 7 min; and from 50% to 80% phase B in 1 min; followed by a 4-min isocratic step at 80% phase B and column re-equilibration. UV detection was at 230 and 270 nm. LC-MS analyses were performed on a Dionex UltiMate 3000 coupled with an LCQ Fleet (Thermo scientific) mass spectrometer equipped with an electrospray interface (ESI) and a tridimensional ion trap. The column was an Atlantis T3 C18 5 μm × 4.6 mm × 50 mm maintained at 40 °C at a flow rate of 0.8 mL/min. Phases A and B were 0.05% TFA in water and acetonitrile, respectively. The elution was with a 14-min multistep program that consisted of 10, 10, 95, 95, 10 and 10% phase B at 0, 1, 7, 12, 12.5 and 14 min, respectively. UV-VIS signals (190–600 nm) were acquired using the diode array detector. The *m/z* range was 110–2000 and the ESI conditions were as follows: spray voltage of 3500 V, capillary temperature of 275 °C, sheath gas flow rate at 35 units and auxiliary gas flow rate at 15 units.

High resolution MS spectra were recorded at Unitech OMICs (University of Milano, Italy) using a Triple TOF^®^ 6600 (Sciex) equipped with an ESI source. The experiments were carried out by direct infusion in positive ionization mode. The ESI parameters were the following: curtain gas 25 units, ion spray voltage floating 5500 v, temperature 50 °C, ion source gas1 10 units, ion source gas2 0 units, declustering potential 80 v, syringe flow rate 10 µL/min, accumulation time 1 s.

Mono- and bi-dimensional NMR spectra were measured in CDCl_3_ at 298K using an AMX 400 MHz spectrometer. Chemical shifts are reported relative to CDCl_3_ (δ 7.26 ppm).

### 3.5. Purification of Polyethers

α-823: Nine parallel 100-mL cultures of *Actinoallomurus* sp. ID145823 in AF-A medium were harvested at seven days and filtered through paper under reduced pressure to separate the mycelium from the clear broth. The latter (860 mL) was extracted three times with 450 mL ethyl acetate while the mycelium was treated with 100 mL acetone, kept on a rotary shaker 1 h and centrifuged. The combined organic phases were dried under reduced pressure and dissolved in 2 mL dichloromethane. The sample was resolved on a 12 g direct-phase Flash column RediSep RF (Teledyne Isco) by using a CombiFlash RF Teledyne Isco medium-pressure chromatography system. The column was previously conditioned at 100% dichloromethane and then eluted at 15 mL/min with a 20-min linear gradient from 0 to 10% methanol. Fractions were analyzed by LC-MS and those with the highest purity were pooled and dried, obtaining 14 mg of purified α-823. Five mg were dissolved in CDCl_3_ for NMR analysis.

Octacyclomycin: Two parallel 100-mL cultures of *Actinoallomurus* sp. ID145817 in AF-A medium were harvested at seven days and filtered through paper under reduced pressure to separate the mycelium from the clear broth. The latter (170 mL) was extracted three times with 80 mL ethyl acetate while the mycelium was treated with 100 mL ethanol, kept 1 h on a rotary shaker and centrifuged. The combined organic phases were dried under reduced pressure and dissolved in 2 mL dichloromethane. The sample was resolved by medium-pressure chromatography as described above for α-823. Fractions were analyzed by LC-MS and processed as above. Four mg of purified octacyclomycin were obtained.

α-770: Two parallel 100-mL cultures of *Actinoallomurus* sp. ID145770 in medium M8 [41] were harvested at seven days. Mycelium was harvested by centrifugation (10 min at 4000 rpm), treated with 20 mL ethanol, kept 1 h on a rotary shaker and centrifuged. The organic phase was recovered, dried under reduced pressure, dissolved in 2 mL dichloromethane and resolved by medium-pressure chromatography as described for α-823, except that the flow rate was set at 30 mL/min. Fractions 11–14, which showed activity against *S. aureus*, were analyzed by LC-MS and the ones containing similar signals were pooled, dried and dissolved in dichloromethane. A further purification step was performed by preparative thin layer chromatography on silica gel (Analtech Preparative Silica Gel GF with UV254 2000 μm; Sigma-Aldrich, St Louis, MO, USA) in dichloromethane:methanol 9:1. The spot at R_f_ 0.9 was dried and dissolved in CDCl_3_ for NMR analysis. Four mg of purified α-770 were obtained.

## 4. Conclusions

When grown under one routine condition in shake-flasks and only looking at metabolites with growth inhibitory activity towards *S. aureus*, we have been able to show that *Actinoallomurus* strains can express several types of biosynthetic pathways: type I (for making polyethers and spirotetronates), type II (for aromatic polyketides) and type III (for the 6-methylsalicylate moiety of the polyether α-770) polyketide synthases; ribosomally synthesized and post-translationally modified peptides (lantibiotic); aminocoumarins; and short non-ribosomal peptide synthase derived products (diketopiperazines).

Some of the observed metabolites, e.g., the previously reported aromatic polyketide allocyclinones [17] and the polyether α-823, seem to be relatively frequent metabolites in the screened *Actinoallomurus* strains. Other metabolites represent rarer discovery events, with identical or close matches in several *Actinobacteria* genera. Indeed, the genus *Actinoallomurus* resulted from a reclassification of *Actinomadura* spp. within the family *Thermomonosporaceae*, order *Streptosporangiales* [9]. Some of the compounds described here (e.g., pradimicin and benanomicin) and the α-770- and α-823-related polyethers cationomycin and SF-2361, respectively, were previously reported from *Actinomadura* spp. Others of the described metabolites are produced by distantly related taxa, such as NAI-107 by *Microbispora* spp. (family *Streptosporangiaceae*, order *Streptosporangiales*), pyrrolosporin by a *Micromonospora* sp. (order *Micromonosporales*), in addition to the *Streptomyces*-produced coumermycin and octacyclomycin.

## Figures and Tables

**Table 1:** Geographic origin of the analyzed strains.

Continent	Analyzed Strains	Active Strains	Active (%)
Europe	124	59	48%
Africa ^a^	24	17	71%
Asia ^a^	12	3	25%
Americas ^b^	40	25	62%

^a^ All from tropical countries; ^b^ All from tropical countries, except four strains from continental USA.

**Table 2:** Polyether-producing *Actinoallomurus* strains.

Strain	Origin	Accession Number ^a^	*m*/*z* [M−Na]^+^	Compound
ID145265	soil, Nicaragua	MH3933000	937	α-823
ID145554	soil, Mauritius	MH3933001	937	α-823
ID145603	soil, Brazil	MH3933002	937	α-823
ID145770	soil, Niger	MH3933011	857	α-770
ID145802	soil, Nicaragua	MH3933003	937	α-823
ID145804	soil, Cameroon	MH3933004	937	α-823
ID145811	soil, Cameroon	MH3933005	937	α-823
ID145816	soil, Cameroon	MH3933006	937	α-823
ID145817	soil, Cameroon	MH3933010	1039	octacyclomycin
ID145823	soil, Venezuela	MH3933007	937	α-823
ID145828	soil, Nicaragua	MH3933008	937	α-823
ID145830	soil, Nicaragua	MH3933009	937	α-823

^a^ On the basis of the 16S rRNA gene sequence.

**Table 3:** MICs (Minimal Inhibitory Concentrations) of α-770 and α-823 in comparison with salinomycin.

Microorganism ^a^	Code	MIC (µg/mL)		
		α-770	α-823	Salinomycin
*Staphylococcus aureus* (MSSA)	ATCC 6538P	0.125	0.5	0.25
*S. aureus* (MRSA)	L1400	0.125	2	0.5
*S. aureus* (GISA)	L3797	0.125	1	0.125
*Streptococcus pyogenes*	L49	≤0.03	≤0.03	≤0.03
*S. pneumoniae*	L44	≤0.03	0.125	0.125
*S. haemolyticus*	L1730	0.125	1	0.5
*S. epidermidis*	ATCC 12228	0.125	1	0.5
*Enterococcus faecalis*	L559	≤0.03	0.25	≤0.03
*E. faecium*	L568	0.25	1	1
*Bacillus subtilis*	ATCC 6633	≤0.03	0.125	0.125
*Micrococcus luteus*	ATCC 10240	0.125	0.5	0.5
*Mycobacterium smegmatis*	mc^2^ 155	16	2	64
*Moraxella catarrhalis*	L3292	2	8	16
*Clostridium difficile*	L4013	0.5	0.125	0.25
*Candida albicans*	L145	>64	>64	>64

^a^ abbreviations: MRSA, methicillin-resistant *Staphylococcus aureus*; MSSA, methicillin-sensitive *Staphylococcus aureus*; GISA, glycopeptide-intermediate *Staphylococcus aureus*.

## Supplementary Materials

The following are available online at http://www.mdpi.com/2079-6382/7/2/47/s1, Figure S1: LC-MS, UV analysis of ethyl acetate extract of *Actinoallomurus* sp. ID145519, Figure S2: LC-MS, UV analysis of ethyl acetate extract of *Actinoallomurus* sp. ID145814, Figure S3: LC-MS, UV analysis of mycelium extract of *Actinoallomurus* sp. ID145640, Figure S4: Comparison between mycelium extract of *Actinoallomurus* sp. ID145640 and NAI-107 standard, Figure S5: LC-MS, UV analysis of ethyl acetate extract of *Actinoallomurus* sp. ID145219, Figure S6: LC-MS, UV analysis of ethyl acetate extract of *Actinoallomurus* sp. ID145114, Figure S7: Fragmentation patterns of the eight different congeners of α-823, Figure S8: 1H-NMR spectrum of α-823, Figure S9: 1H-COSY NMR spectrum of α-823, Figure S10: 1H-TOCSY NMR spectrum of α-823, Figure S11: 1H-13C HSQC NMR spectrum of α-823, Figure S12: 1H-13C HMBC NMR spectrum of α-823, Figure S13: Major COSY and HMBC correlations of α-823, Figure S14: HRESI-MS spectrum of α-823, Figure S15: LC-MS, UV analysis of mycelium extract of *Actinoallomurus* sp. ID145817, Figure S16: Fragmentation patters of the two different congeners of α-770, Figure S17: 1H NMR spectrum of α-770, Figure S18: 1H-COSY NMR spectrum of α-770, Figure S19: 1H-TOCSY NMR spectrum of α-770, Figure S20: 1H-13C HSQC NMR spectrum of α-770, Figure S21: 1H-13C HMBC NMR spectrum of α-770, Figure S22: Major COSY and HMBC correlations of α-770, Figure S23: HRESI-MS spectrum of α-770, Table S1: Different α-823 congeners detected in the extract from *Actinoallomurus* sp. ID145823, Table S2: 1H and 13C NMR data for α-823 in CDCl3, Table S3: α-770 congeners detected in the extract from *Actinoallomurus* sp. ID145770, Table S4: 1H and 13C NMR data for α-770 in CDCl3.
