# Supplementary material for: Novel Polyethers from Screening Actinoallomurus spp
Source: Antibiotics (Basel). 2018 Jun 14;7(2):47. doi: 10.3390/antibiotics7020047 (PMC6023020; doi:10.3390/antibiotics7020047)
Supplement: Supplementary file 1 [file antibiotics-07-00047-s001.pdf]

Supplementary Material

Novel polyethers from screening *Actinoallomurus* spp.

Marianna Iorio, Arianna Tocchetti, Joao Carlos Santos Cruz, Giancarlo Del Gatto, Cristina Brunati, Sonia Ilaria Maffioli, Margherita Sosio, Stefano Donadio

Table S1. The α-823 congeners detected in the extract from *Actinoallomurus* sp. ID145823.

Congener	*m/z* [M+Na]$^+$	RT (min)	Δ *m/z*	Molecular formula	calc. *m/z*	found *m/z*
α-823 A	937	8.1	---	$C_{48}H_{82}O_{16}Na$	937.5495	937.5510
α-823 B	893	7.3	-44	$C_{47}H_{82}O_{14}Na$	893.5597	893.5589
α-823 C	937	7.0	---	$C_{48}H_{82}O_{16}Na$	937.5495	937.5510
α-823 D	923	6.1	-14	$C_{47}H_{80}O_{16}Na$	923.5339	923.5376
α-823 E	951	5.5	+14	$C_{49}H_{84}O_{16}Na$	951.5652	951.5611
α-823 F	937	4.6	---	$C_{48}H_{82}O_{16}Na$	937.5495	937.5510

Table S2. ^{1}H and ^{13}C NMR data for *α*-823 in $CDCl_3$.

Position	δH, (*J* in Hz)	δC, type
1		180.3, C
2	2.18 (*) - 2.54, d (12.6)	44.7, CH2
3		97.9, C
4	1.51 (*)	44.7, CH
Me (4)	1.04, d (6.6)	12.5, CH3
5	3.42, d (10.2)	84.4, CH
OMe (5)	3.57, s	60.5, CH3
6		78.6, C
OMe (6)	3.38, s	50.0, CH3
Me (6)	1.16, s	11.4, CH3
7	3.90, m	66.0, CH
8	1.58 (**)	32.6, CH2
9	4.08, m	60.9, CH
10	1.16 (*) - 2.21 (*)	31.3, CH2
11	3.39	79.5, CH
OMe (11)	3.53, s	59.0, CH3
12	1.84	33.6, CH
Me (12)	0.96, d (7.5)	12.5, CH3
13		108.2, C
14	2.47, m	39.0, CH
Me (14)	0.94, d (6.2)	12.1, CH3
15	1.47 (*) - 2.38 (*)	38.7, CH2
16		82.7, C
Me (16)	1.49, s	27.9, CH3
17	3.69, d (6.3)	87.4, CH
18	4.42, m	74.1, CH
19	1.47 (*) - 2.19 (*)	36.6, CH2
20		83.9, C
Me (20)	1.24, s	22.0, CH3
21	4.24, dd (5.3, 11.0)	83.4, CH
22	1.88 (*) - 2.03 (m)	29.7, CH2
23	1.81 (*)- 2.21 (*)	24.2, CH2
24	4.32, m	80.8, CH
25	3.86, m	74.8, CH
26	1.36, m	33.0, CH
Me (26)	0.84, d (7.1)	17.4, CH3
27	1.47, m	36.0, CH2
28	1.53 (**)	39.6, CH
Me (28)	0.93, d (6.2)	16.9, CH3
29		96.5, C
Me (29)	1.33, s	26.5, CH3
1′	4.44, m	99, CH
2′	1.51 (*) - 1.84(*)	30.7, CH2
3′	1.36 (*) - 2.24 (*)	26.9, CH2
4′	2.81,dt (4.8, 10.2)	79.0, CH
OMe (4′)	3.38, s	56.7, CH3
5′	3.29, m	74.5, CH
Me (5′)	1.26, d (6.6)	18.0, CH3

*signals overlapped; ** under H_2O signal

Table S3. α-770 congeners detected in the extract from *Actinoallomurus* sp. ID145770.

Congener	*m/z* [M+Na]$^+$	RT (min)	Δ *m/z*	Molecular formula	calc. *m/z*	found *m/z*
α-770 A	857	6.9	---	$C_{45}H_{70}O_{14}Na$	857.4658	857.4695
α-770 B	871	7.1	+14	$C_{46}H_{72}O_{14}Na$	871.4814	871.4845

Table S4. ^{1}H and ^{13}C NMR data for α-770 in $CDCl_3$.

Position	δH, (J in Hz)	δC, type
1		180.2, C
2	2.89, m	43.4, CH
Me (2)	1.21, d (6.1)	17.4, CH_3
3	5.56, m (10.6,1.4)	78.4, CH
4	2.14, m	39, CH
Me (4)	1.22 (*)	11.6, CH_3
5	4.19, d (4.1)	67, CH
6	2.56, m	29.4, CH
Me (6)	0.96 (*)	10.7, CH_3
7	3.29 (*)	82.2, CH
OMe (7)	3.56, s	58.9, CH_3
8	1.77 (*) – 1.97 (*)	33.7, CH_2
9		104.5, C
10	1.63 (*) – 2.41dd (12.6, 7.7)	45.9, CH_2
11	3.96, m	87.5, CH
OMe (11)	3.29, s	58.4, CH_3
12		77.5, C
Me (12)	1.70, s	27.8, CH_3
13	3.74, m	77.8, CH
14	1.58 (*) – 1.96 (*)	26.5, CH_2
15	1.45 (*) – 2.04 (*)	29.8, CH_2
16		82.7, C
Me (16)	1.12, s	21.5, CH_3
17	4.18, m	85.1, CH
18	2.34, m	35.1, CH
Me (18)	0.92 (*)	15.2, CH_3
19	1.60 (*) – 2.16 (*)	35.1, CH_2
20	4.28, m	78.1, CH
21		106.4, C
22	1.92 (*)	38.9, CH
Me (22)	0.97 (*)	13.2, CH_3
23	1.54 (*) – 1.97 (*)	35.3, CH_2
24		86.7, C
Me (24)	1.19, s	24.9, CH_3
25	3.57 (*)	74.9, CH
26	1.09 (*) – 1.39 (*)	26.2, CH_2
27	0.93 (*)	10.6, CH_3
1′		171.4, C
2′		112.4, C
3′		163.1, C
4′	6.85, d (8)	115.7, CH
5′	7.27, (*)	133.8, CH
6′	6.72, d (7.5)	122.7, CH

*signals overlapped

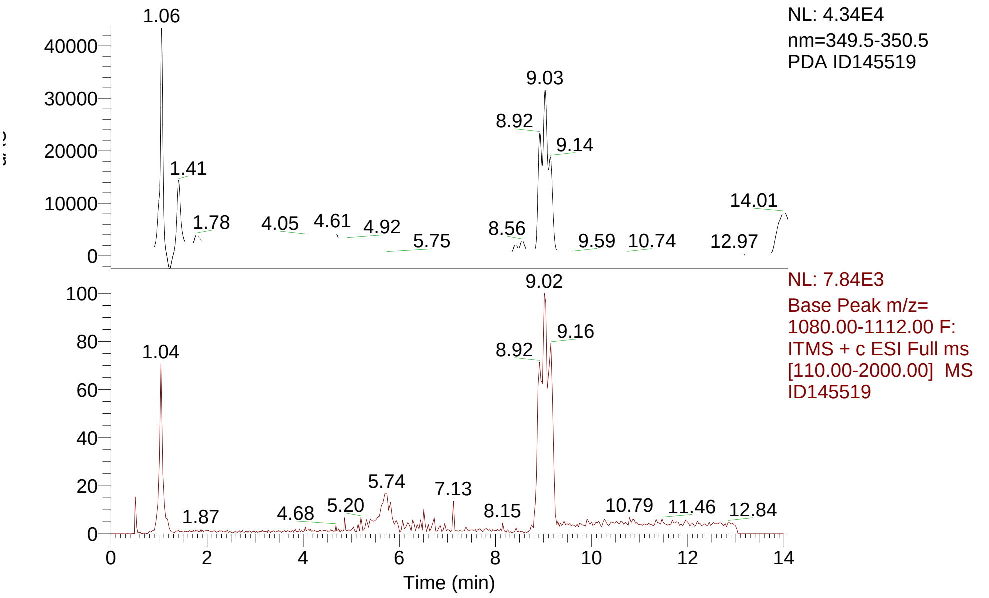

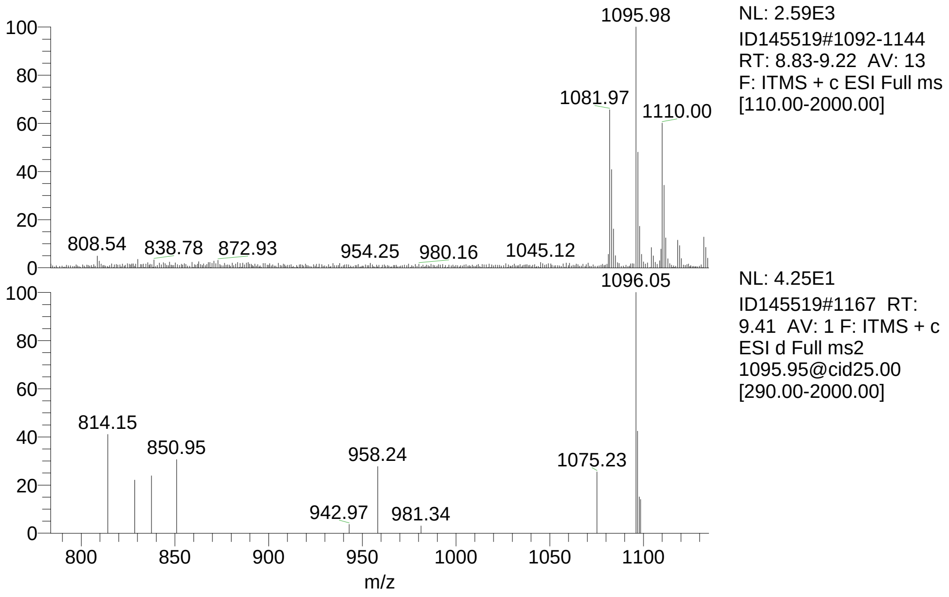

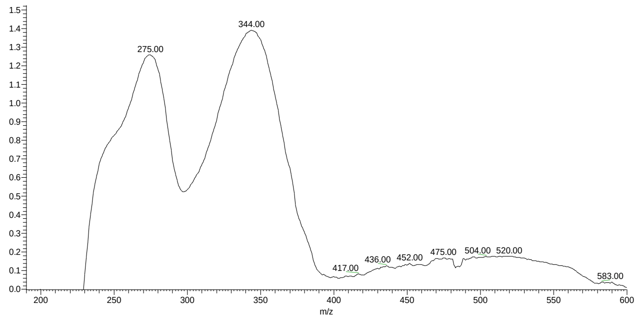

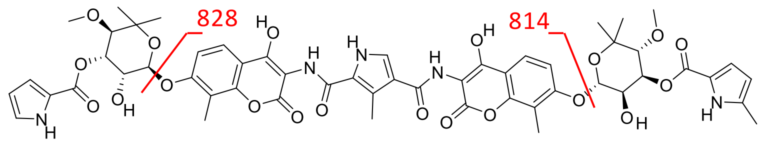

Coumermycin D1

Figure S1. Ethyl acetate extract of *Actinoallomurus* sp. ID145519. From top to bottom: UV chromatogram at 350 nm; extracted ion chromatogram of *m*/*z* $[M+H]^+$ in the 1080–1110 range; mass spectrum of peaks at 8.8–9.2 min, with *m*/*z* $[M+H]^+$ 1082, 1096 and 1110, corresponding to coumermycin A2, D1 and A1, respectively; MS^2 analysis of *m*/*z* $[M+H]^+$ 1096; and UV spectrum of peak at 8.9 min. The putative fragmentation pathway for coumermycin D1 is shown at the very bottom.

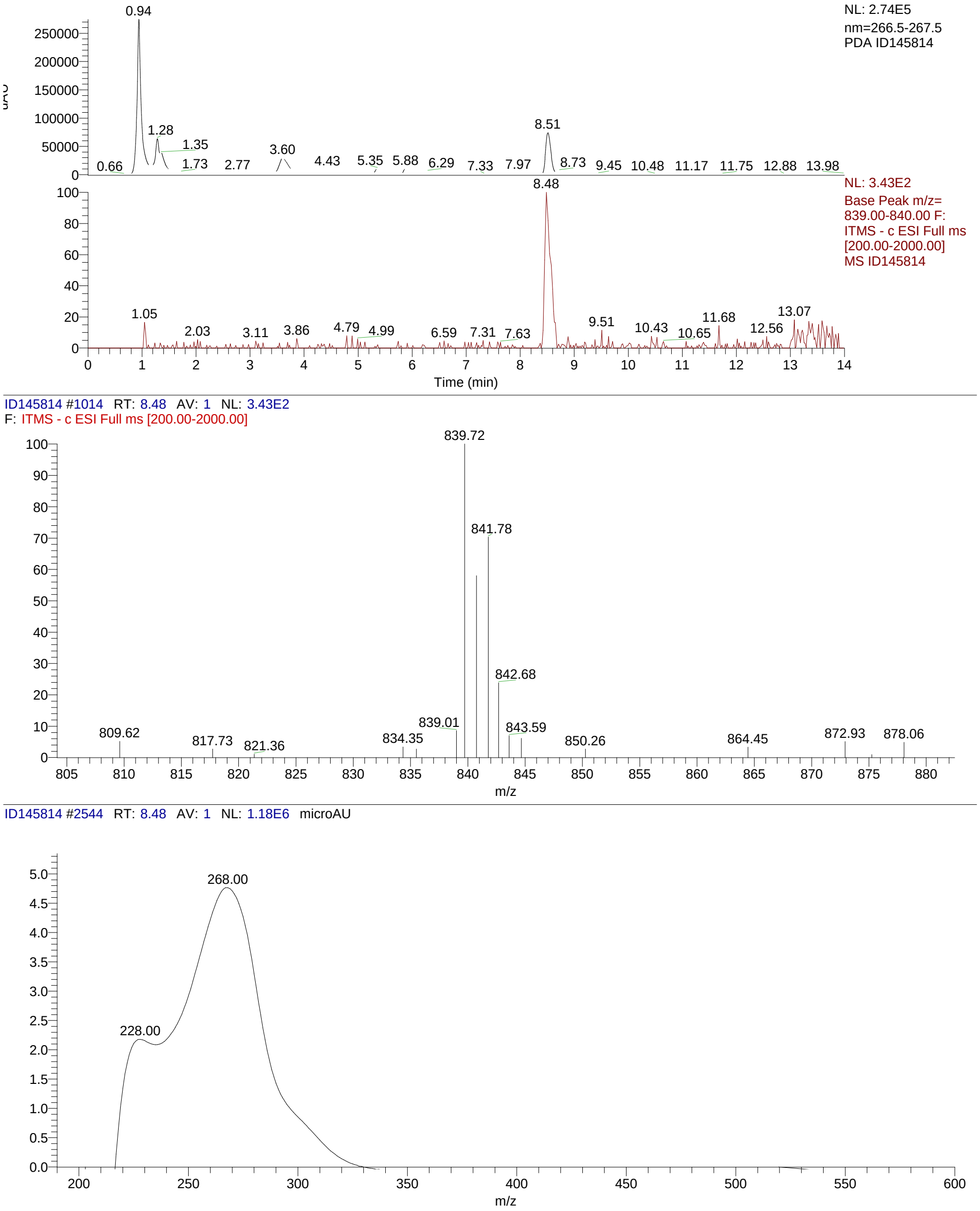

Figure S2. Ethyl acetate extract of *Actinoallomurus* sp. ID145814. From top to bottom: UV chromatogram at 267 nm; extracted ion chromatogram of *m/z* 839 [M-H]$^-$; MS analysis of peak at 8.5 min corresponding to the pyrrolosporin-like molecule (pyrrolosporin A *m/z* 839 [M-H]$^-$); UV spectrum of peak at 8.5 min. The isotopic pattern is compatible with a bis-chlorinated compound and the UV spectrum is identical to that reported in literature for pyrrolosporin A. Note that under our experimental conditions we observed no fragmentation of the 8.5-min peak.

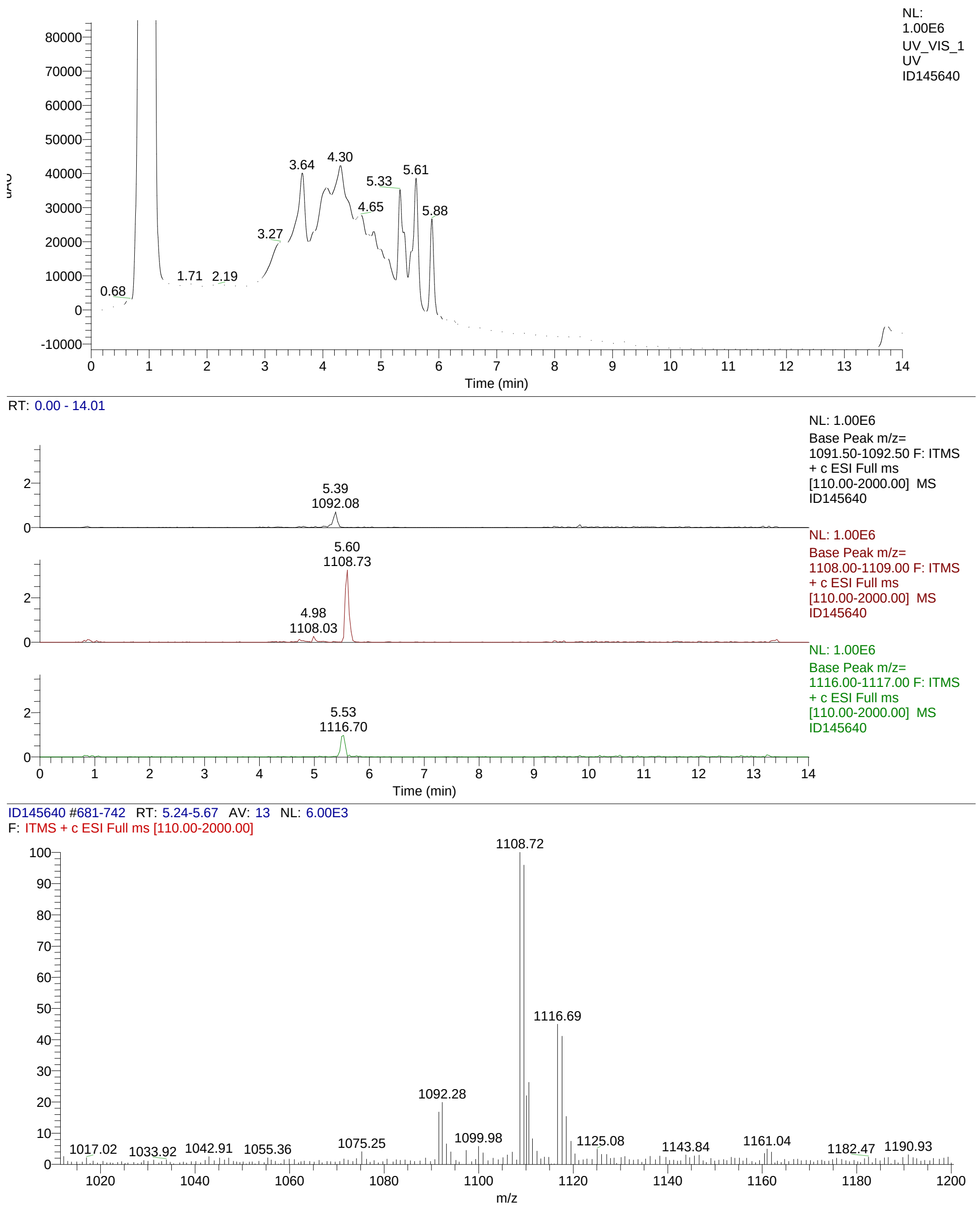

Figure S3. Mycelium extract of *Actinoallomurus* sp. ID145640. From top to bottom: UV chromatogram at 230 nm; extracted ion chromatograms of *m/z* $[M+2H]^{2+}$ 1092, 1108.5 and 1116.5; mass spectrum of peaks at 5.2-5.7 min, with *m/z* $[M+2H]^{2+}$ 1092, 1108.5 and 1116.5, corresponding to NAI-107 F0 (5.4 min), F2 (4.9 min), A0 (5.6 min) and A2 (5.5 min), respectively.

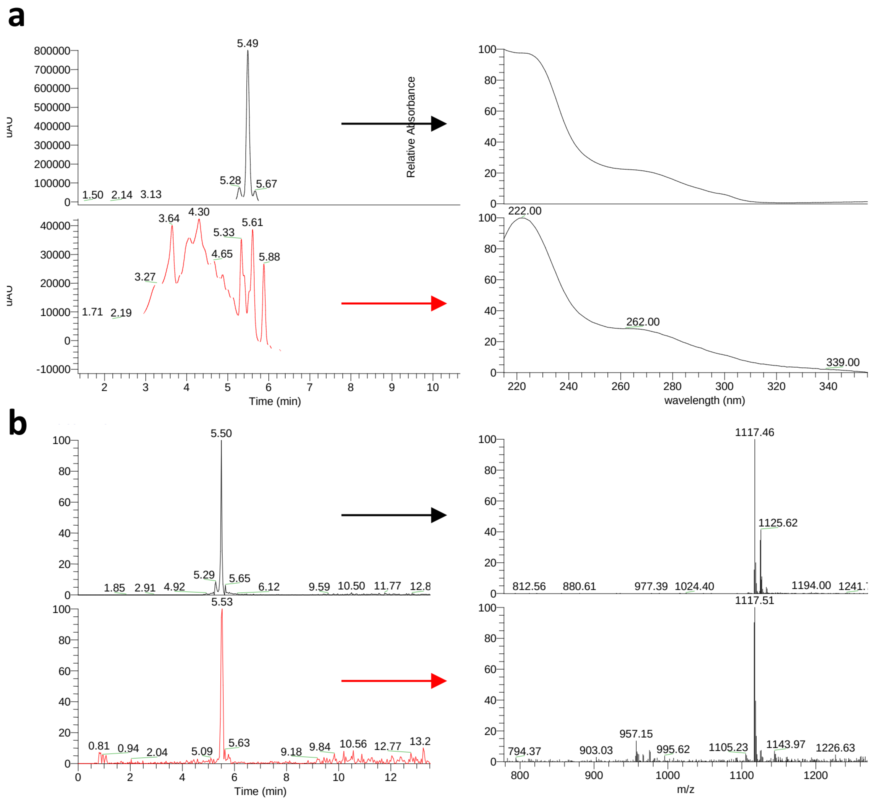

Figure S4. Comparison between LC-MS analyses of NAI-107 standard (black lines) and the mycelium extract of *Actinoallomurus* sp. ID145640 (red lines). Panel a: UV chromatogram at 230 nm with the respective total UV spectra at 5.5 min. Panel b: extracted ion chromatograms of *m/z* $[M+2H]^{2+}$ 1116.5 with the respective MS analysis at 5.5 min, corresponding to NAI-107 A2 representing the main congener present in the purified standard.

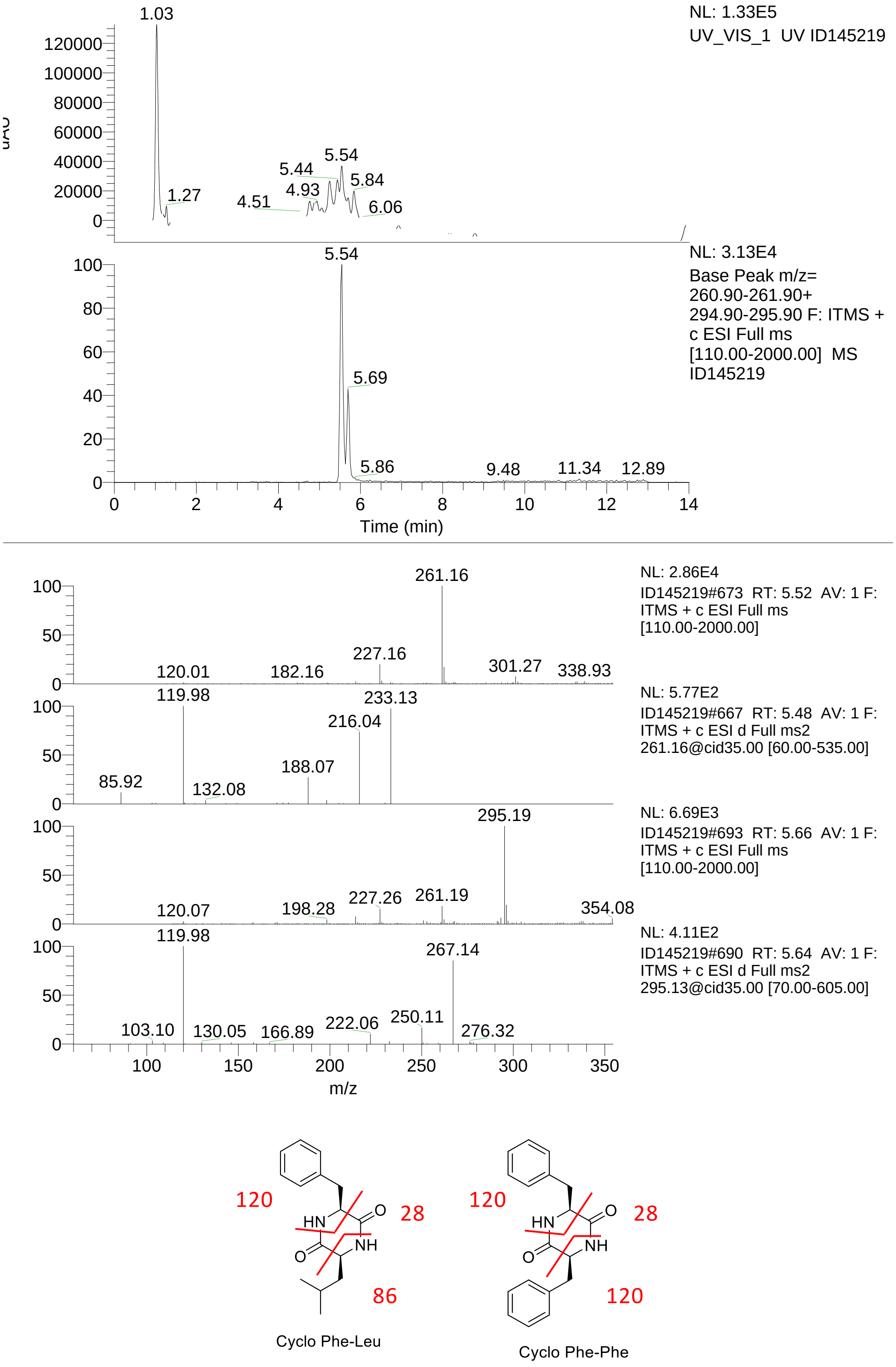

Figure S5. Ethyl acetate extract of *Actinoallomurus* sp. ID145219. From top to bottom: UV chromatogram at 230 nm; extracted ion chromatogram of *m/z* $[M+H]^+$ 261 and 295; MS analysis of peak at 5.5 min, along with its MS^2 data, corresponding to cycloPhe-Leu; and MS analysis of peak at 5.7 min, along with its MS^2 data, corresponding to cycloPhe-Phe. Putative fragmentation pathways for cycloPhe-Leu and cycloPhe-Phe are shown at the bottom.

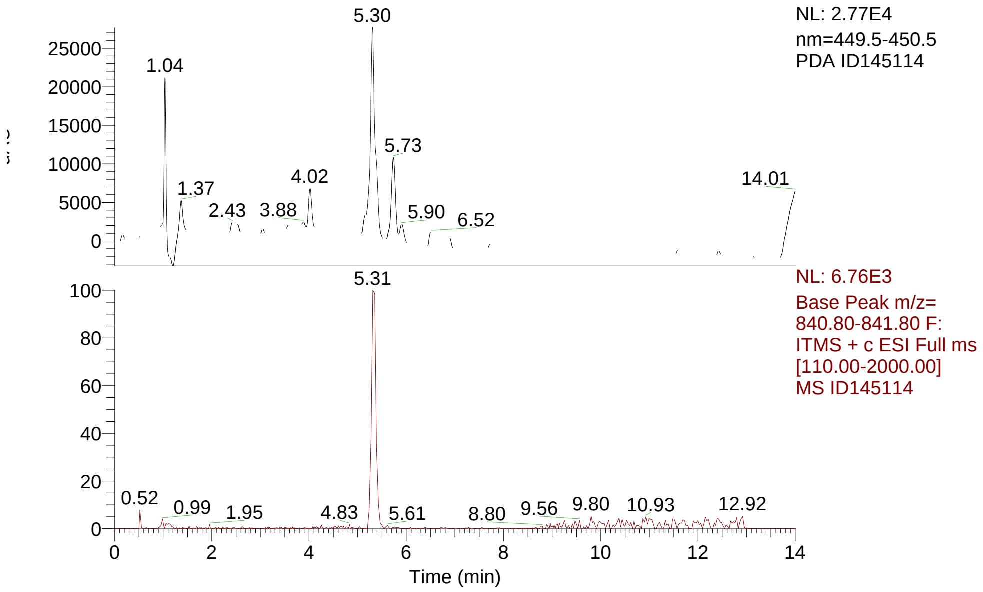
NL: 2.77E4
nm=449.5-450.5
PDA ID145114
25000
20000
15000
10000
5000
0
1.04
1.37
2.43
3.88
4.02
5.30
5.73
5.90
6.52
14.01
NL: 6.76E3
Base Peak m/z=
840.80-841.80 F:
ITMS + c ESI Full ms
[110.00-2000.00]
MS ID145114
100
80
60
40
20
0
0.52
0.99
1.95
4.83
5.31
5.61
8.80
9.56
9.80
10.93
12.92
0
2
4
6
8
10
12
14
Time (min)

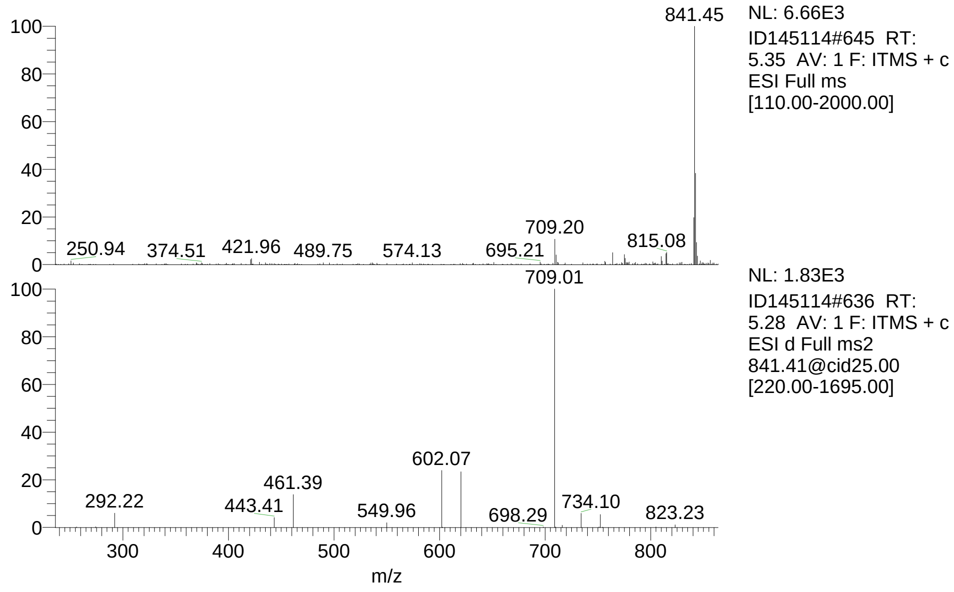
NL: 6.66E3
ID145114#645 RT:
5.35 AV: 1 F: ITMS + c
ESI Full ms
[110.00-2000.00]
100
80
60
40
20
0
841.45
250.94
374.51
421.96
489.75
574.13
695.21
709.20
815.08
NL: 1.83E3
ID145114#636 RT:
5.28 AV: 1 F: ITMS + c
ESI d Full ms2
841.41@cid25.00
[220.00-1695.00]
709.01
602.07
461.39
292.22
443.41
549.96
698.29
734.10
823.23
300
400
500
600
700
800
m/z

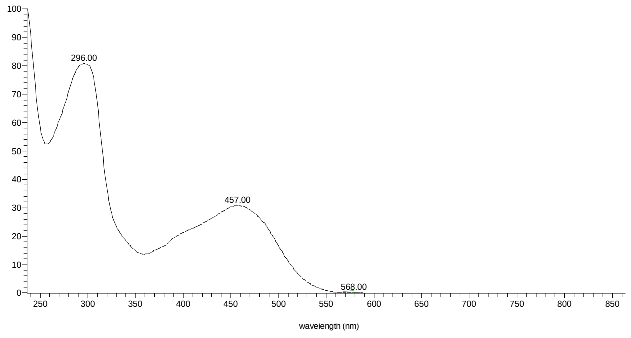
100
90
80
70
60
50
40
30
20
10
0
296.00
457.00
568.00
250
300
350
400
450
500
550
600
650
700
750
800
850
wavelength (nm)

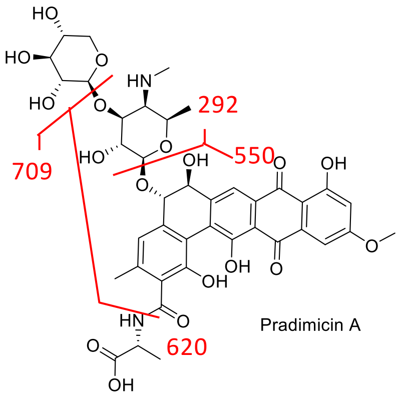

Figure S6. Ethyl acetate extract of *Actinoallomurus* sp. ID145114. From top to bottom: UV chromatogram at 450 nm; extracted ion chromatogram of *m*/*z* $[M+H]^+$ 841; mass spectrum of peak at 5.3 min corresponding to pradimicin (*m*/*z* $[M+H]^+$ 841); MS^2 spectrum of *m*/*z* $[M+H]^+$ 841; and UV spectrum of peak at 5.3 min. The UV maxima are identical to those reported for pradimicin in acidic conditions (see Ref. 28 in the main text). Putative fragmentation pathway for pradimicin. Benanomicin was de-replicated by comparison with the standard obtained from strain K15 strain, as previously (ref. 12 in the main text).

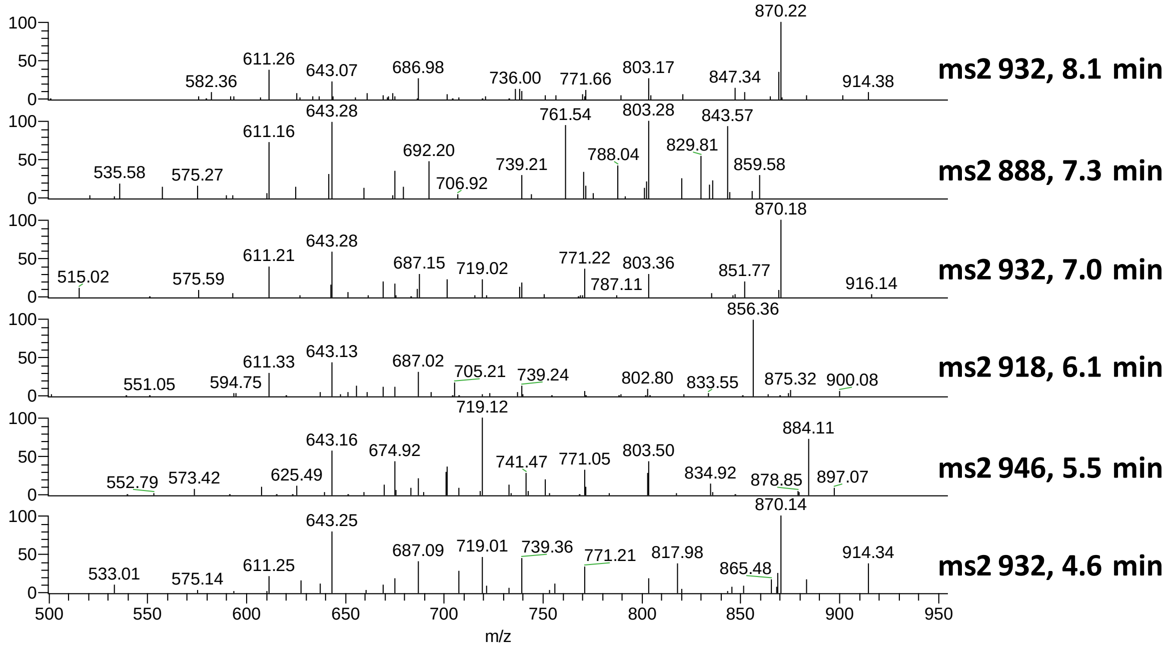

Figure S7. Fragmentation patterns of the $[M+NH_4]^+$ adducts of the six α-823 congeners (see also Table S2).

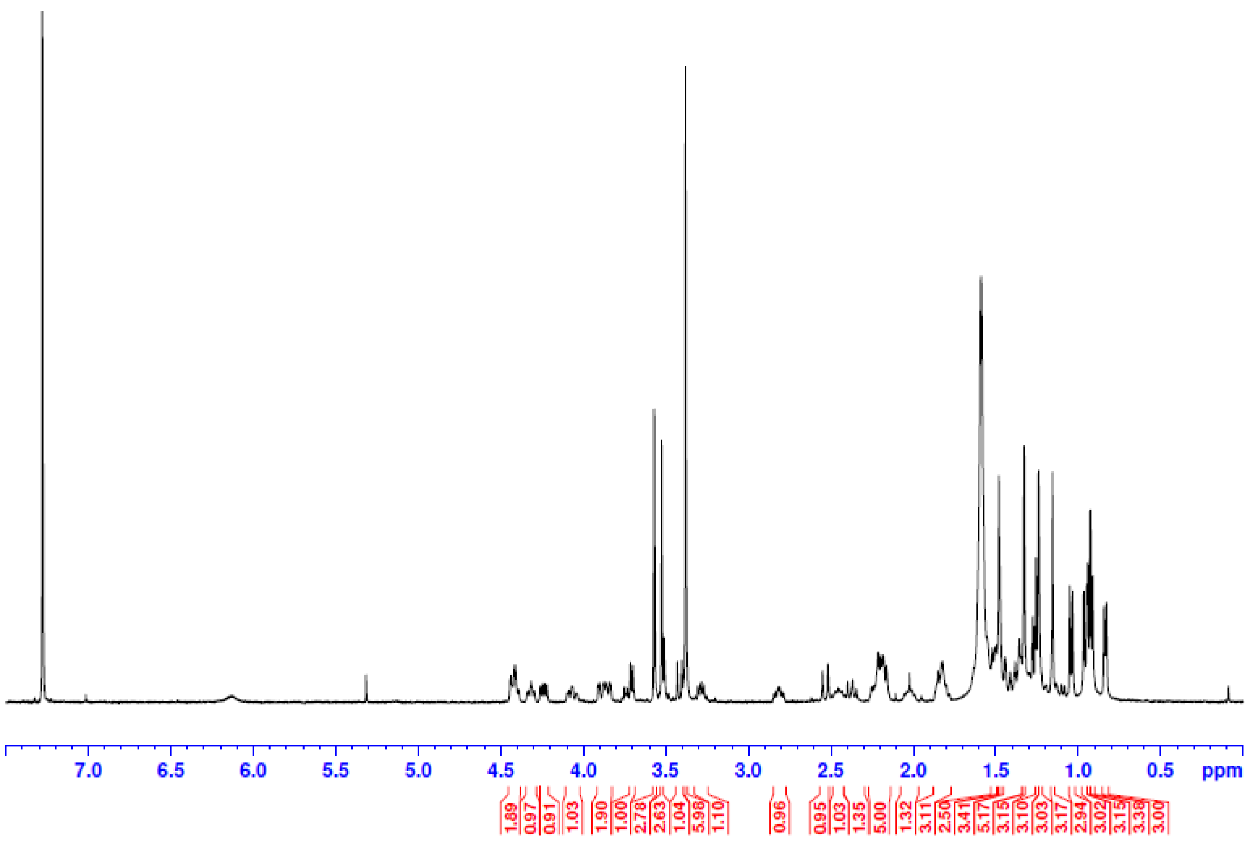

Figure S8. ¹H-NMR (400 MHz, $CDCl_3$) spectrum of α-823.

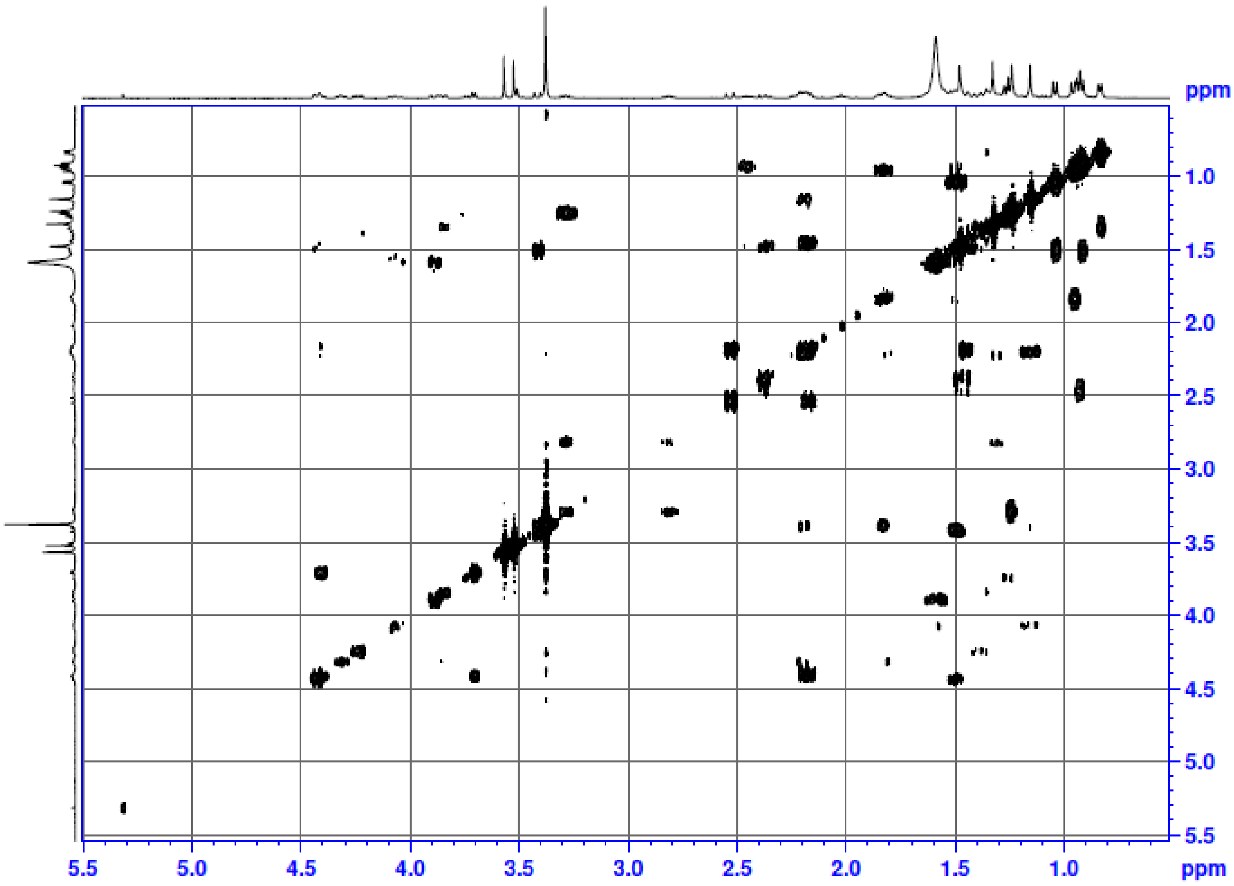

Figure S9. ^{1}H-COSY NMR (400 MHz, $CDCl_3$) spectrum of α-823.

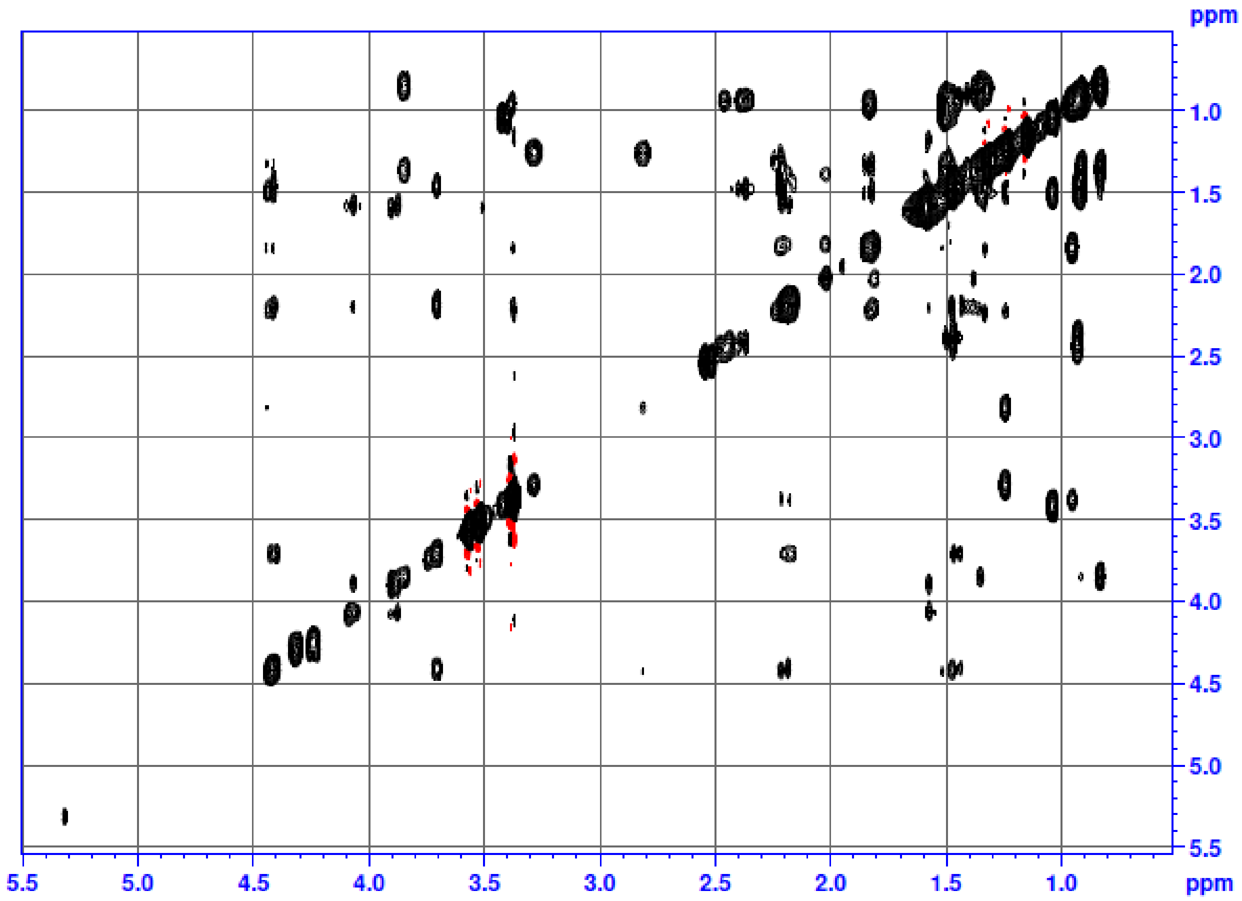

Figure S10. ^{1}H-TOCSY NMR (400 MHz, $CDCl_3$) spectrum of α-823.

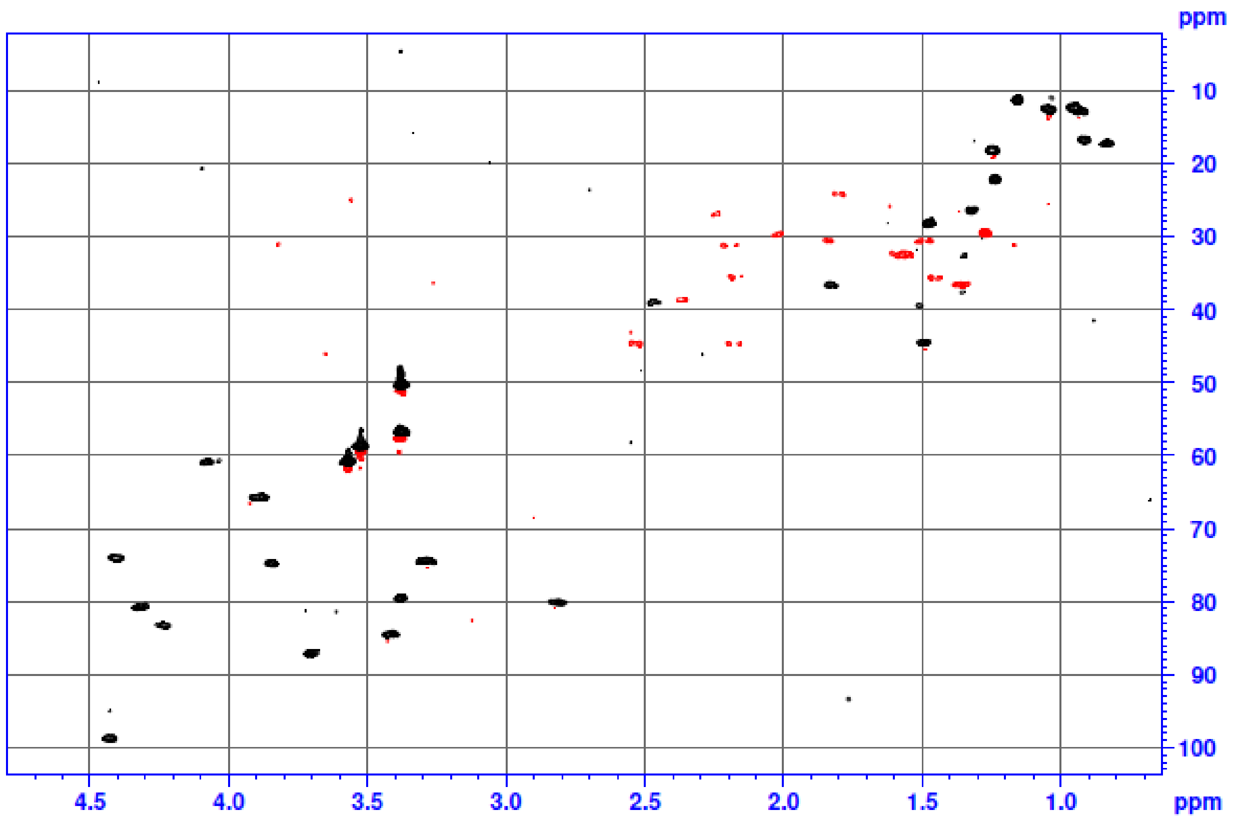

Figure S11. ^{1}H-^{13}C HSQC NMR (400 MHz, $CDCl_3$) spectrum of α-823.

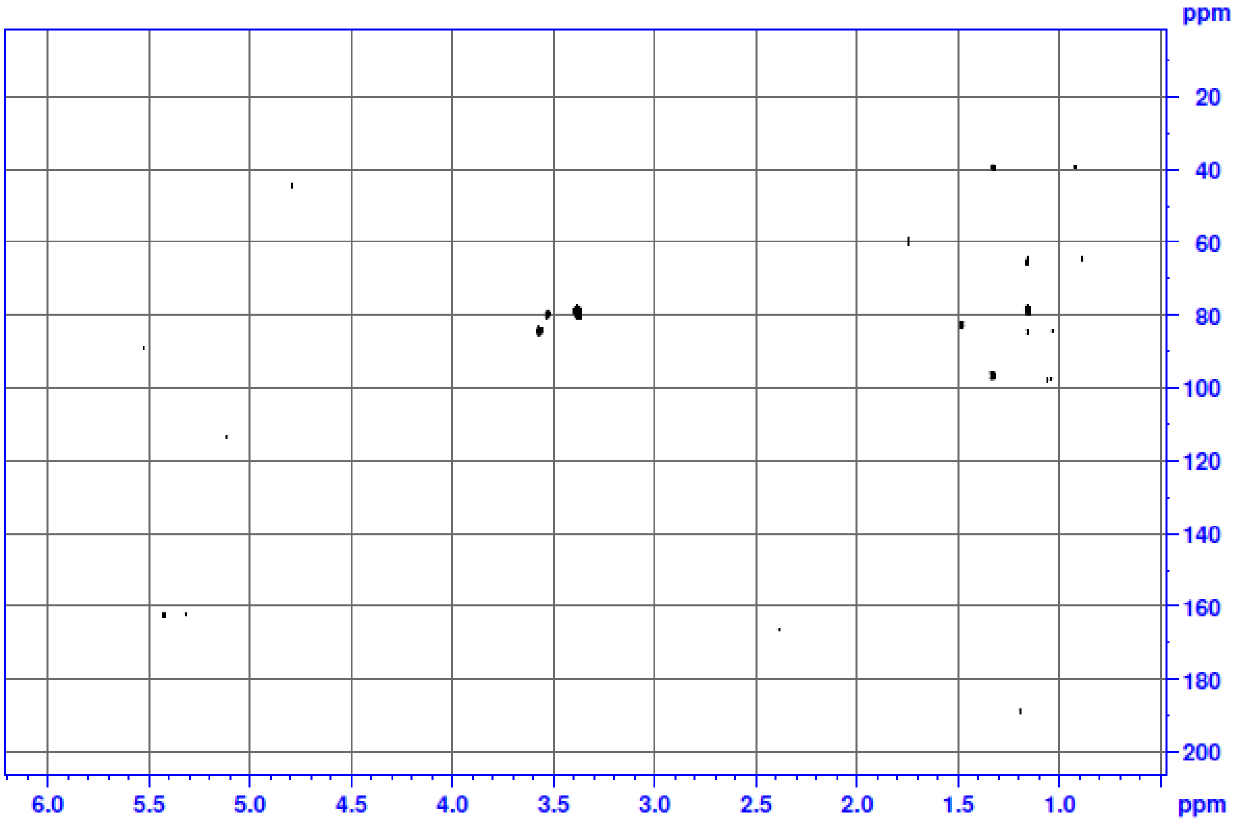

Figure S12. ^{1}H-^{13}C HMBC NMR (400 MHz, $CDCl_3$) spectrum of α-823.

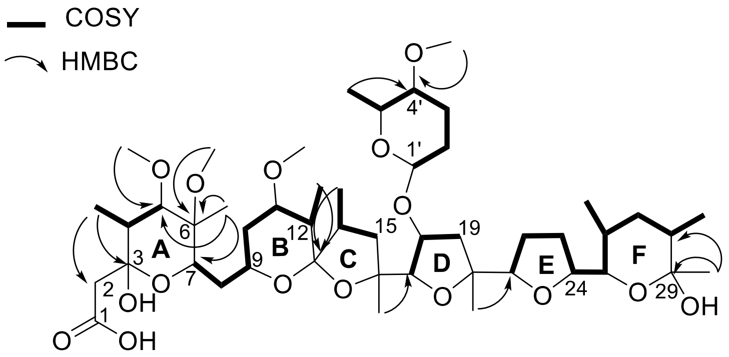

Figure S13. Major COSY and HMBC correlations of *α*-823.

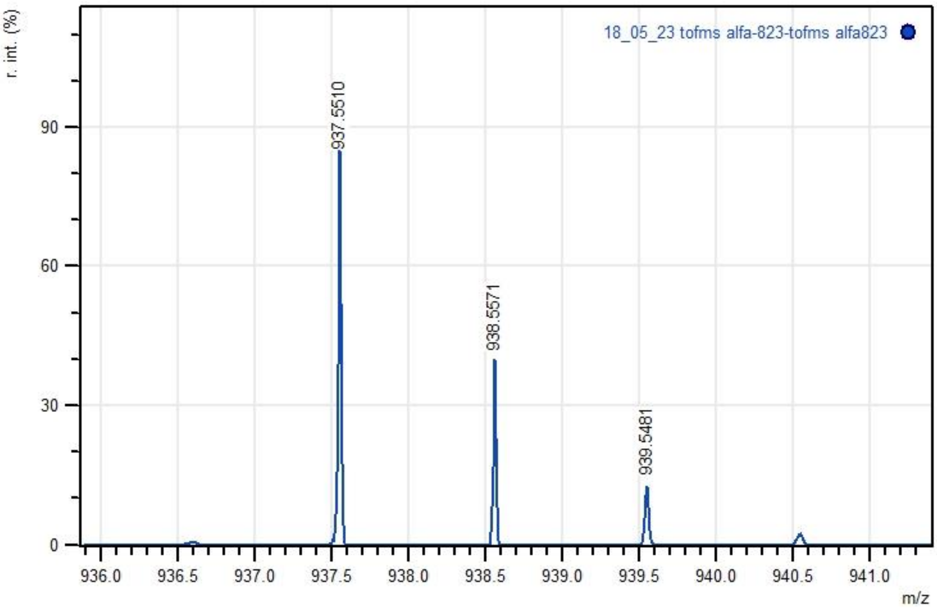

Figure S14. High resolution MS spectrum of α-823.

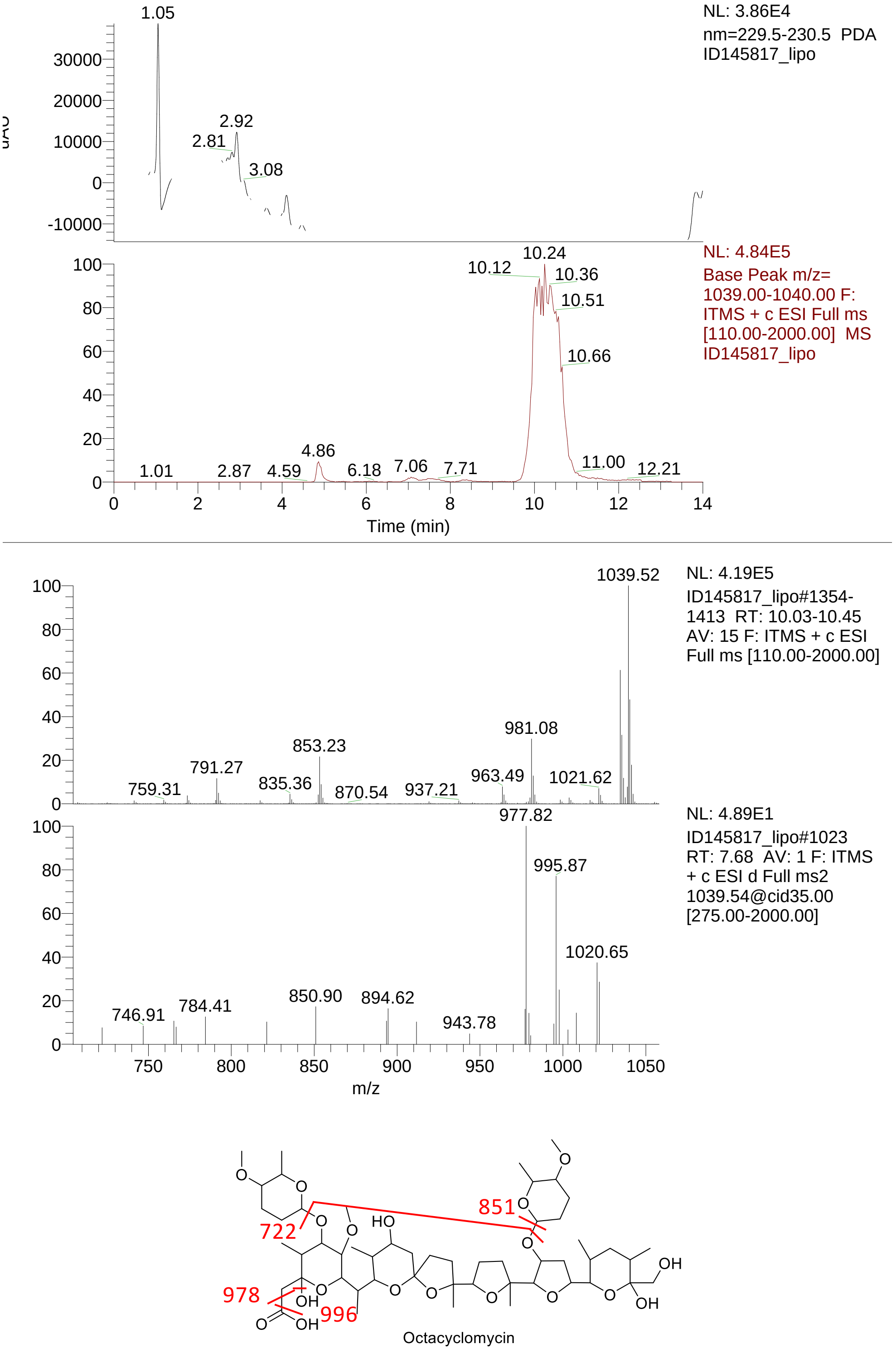

Figure S15. Mycelium extract of *Actinoallomurus* sp. ID145817. From top to bottom: UV chromatogram at 230 nm; extracted ion chromatogram of *m/z* $[M+Na]^+$ 1039; mass spectrum of peak at 10.2 min; and MS^2 analysis of *m/z* $[M+Na]^+$ 1039, corresponding to octacyclomycin. Putative fragmentation pathway for octacyclomycin is shown at the bottom.

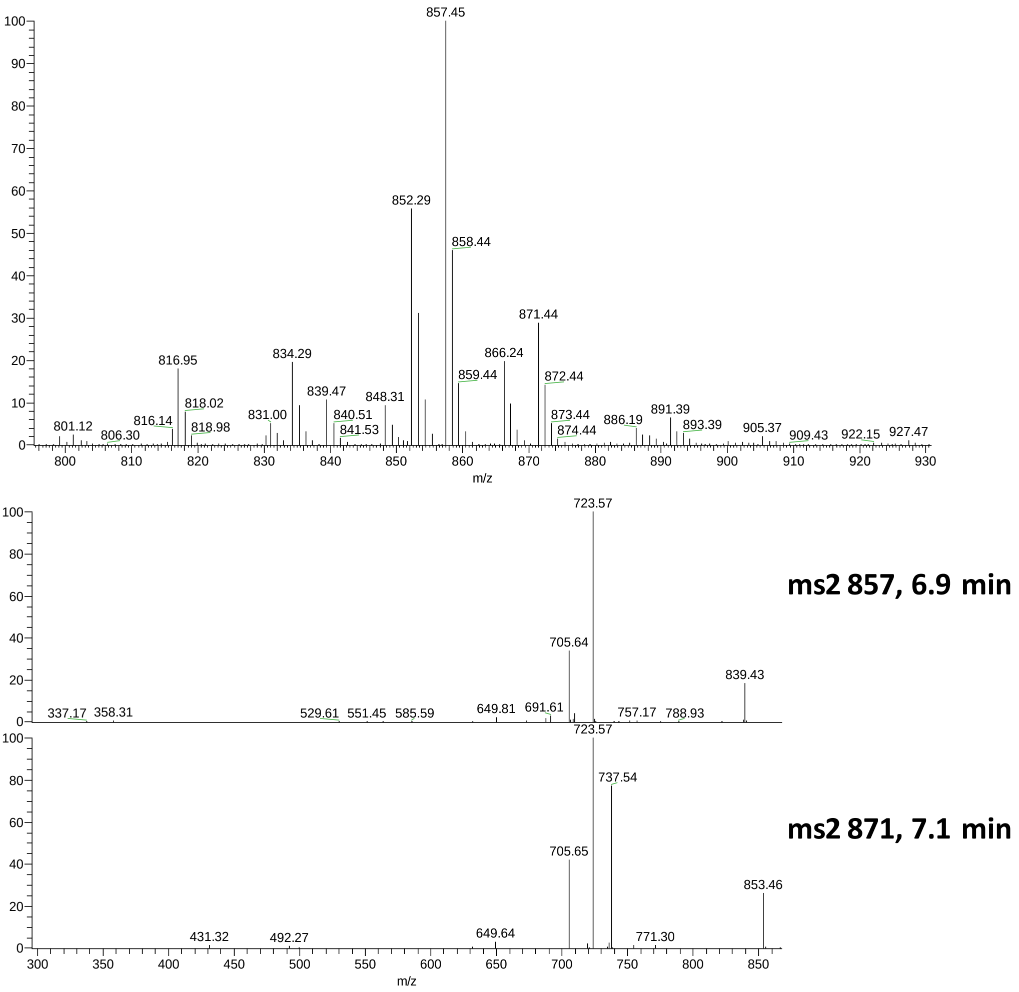

Figure S16. Full MS spectrum between 6.5-7.2 min. Fragmentation patters of the $[M+NH_4]^+$ adducts of the two α-770 congeners of (see also Table S3).

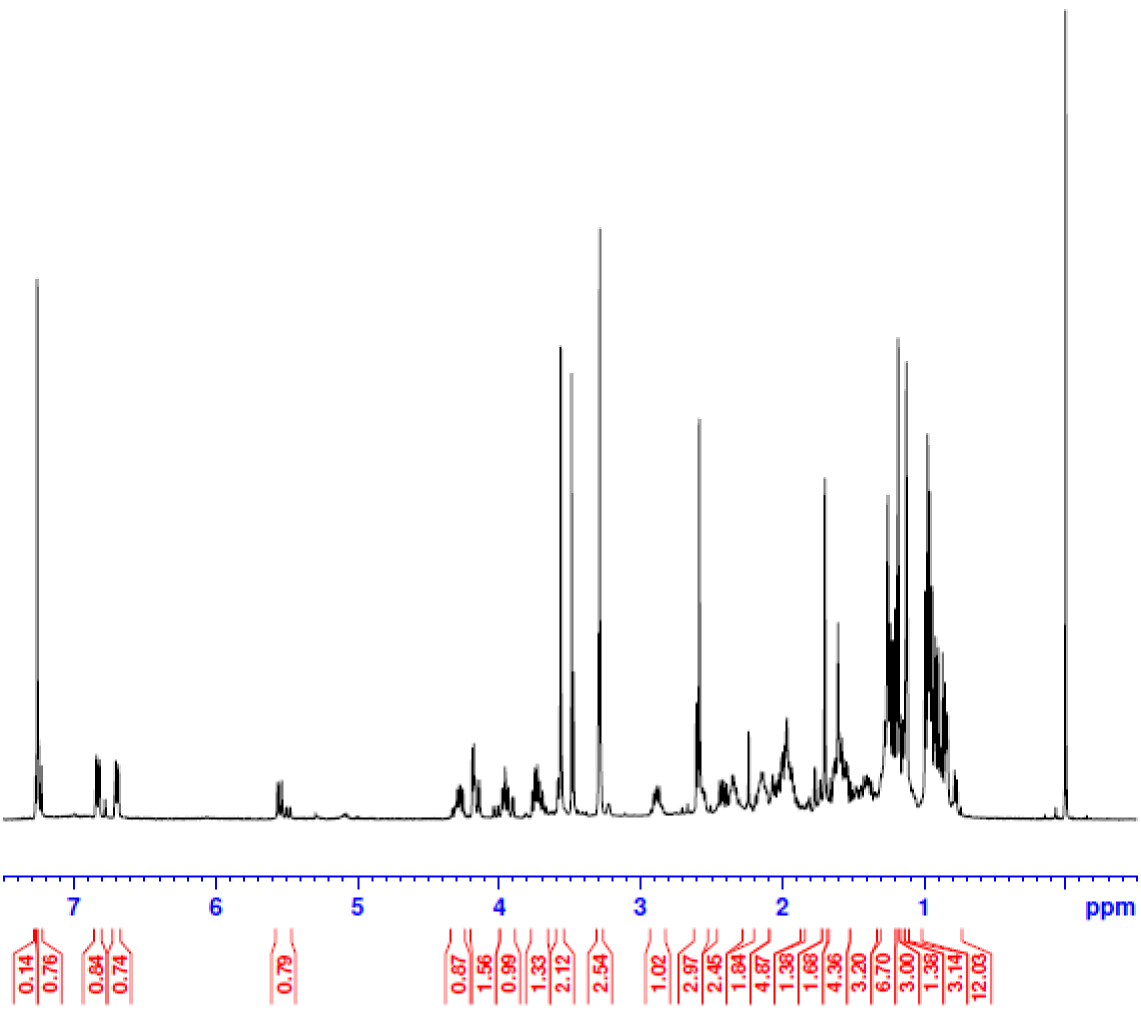

Figure S17. ^{1}H NMR (400 MHz, $CDCl_3$) spectrum of α-770.

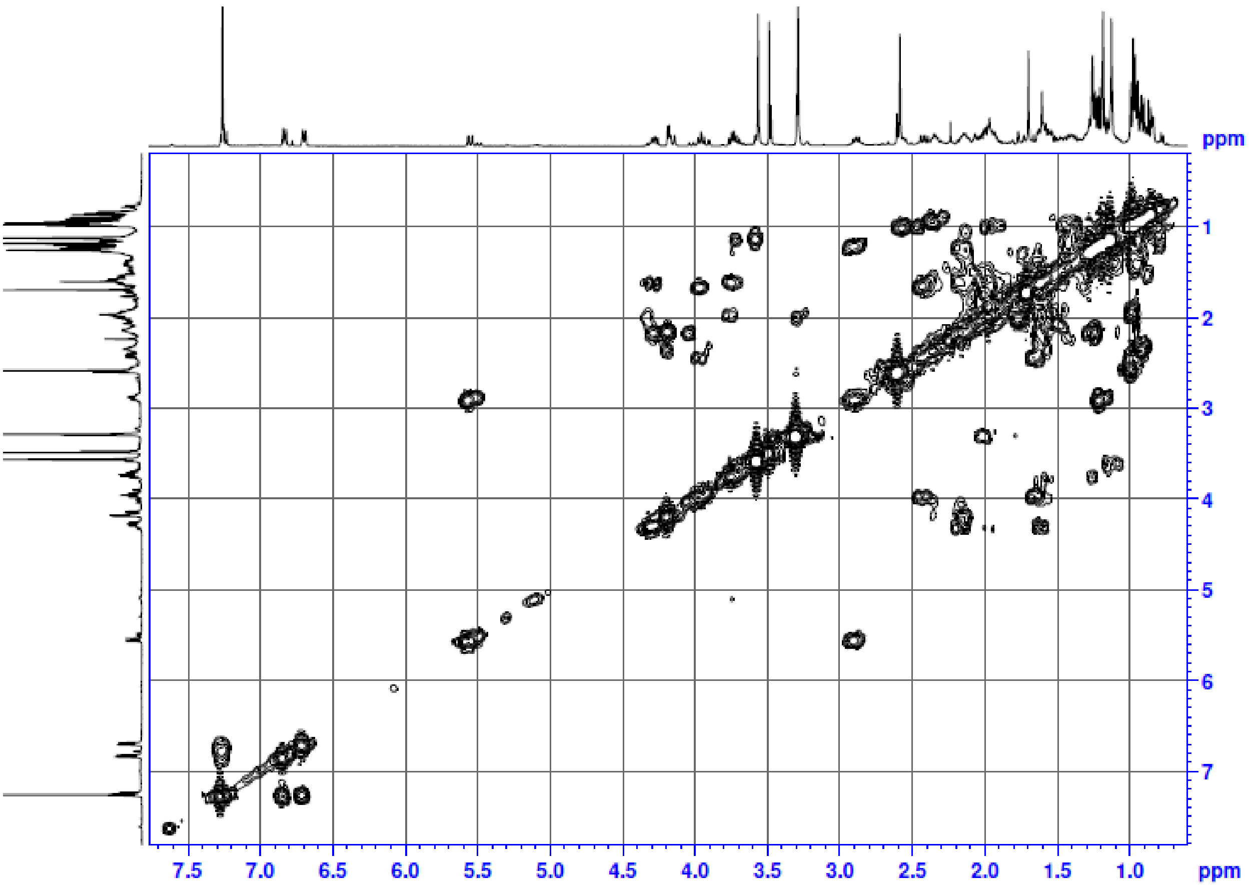

Figure S18. ^{1}H-COSY NMR (400 MHz, $CDCl_3$) spectrum of α-770

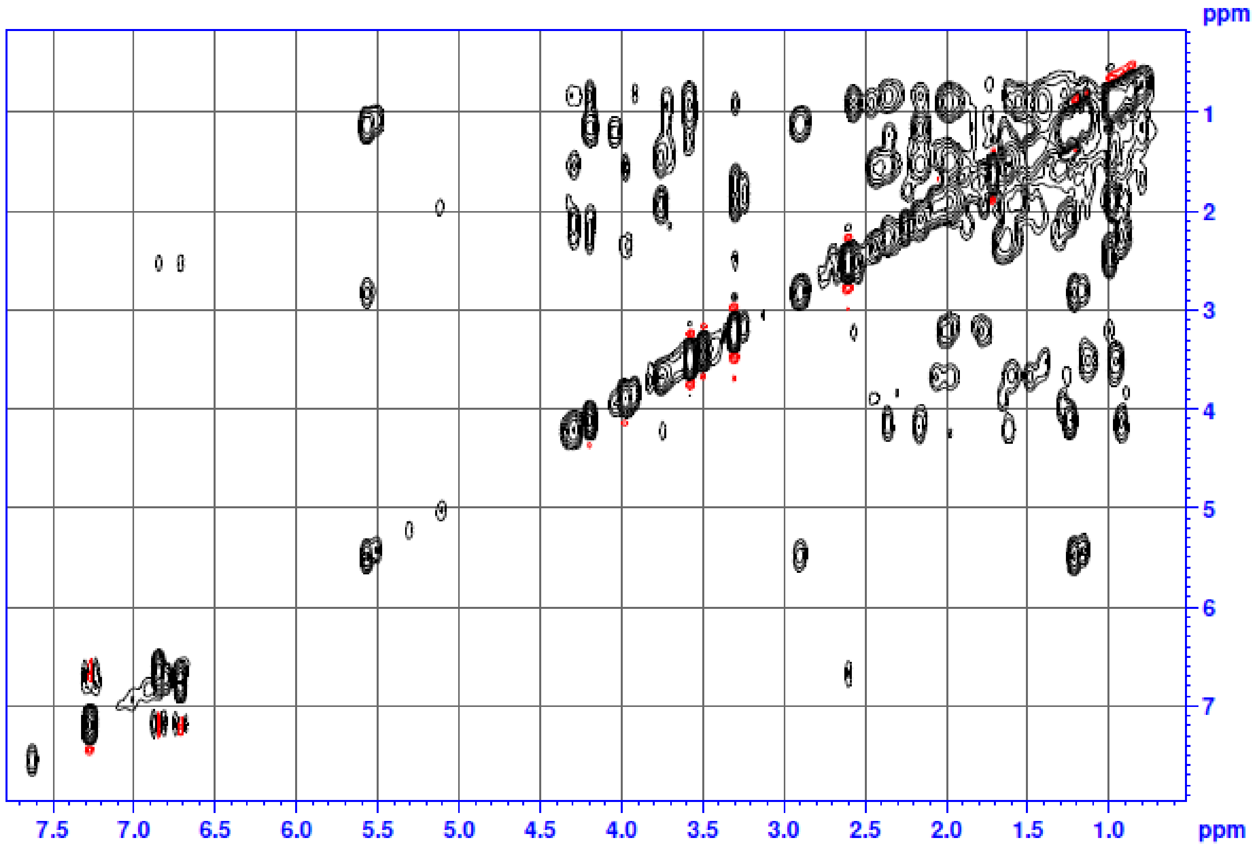

Figure S19. ^{1}H-TOCSY NMR (400 MHz, $CDCl_3$) spectrum of *α*-770.

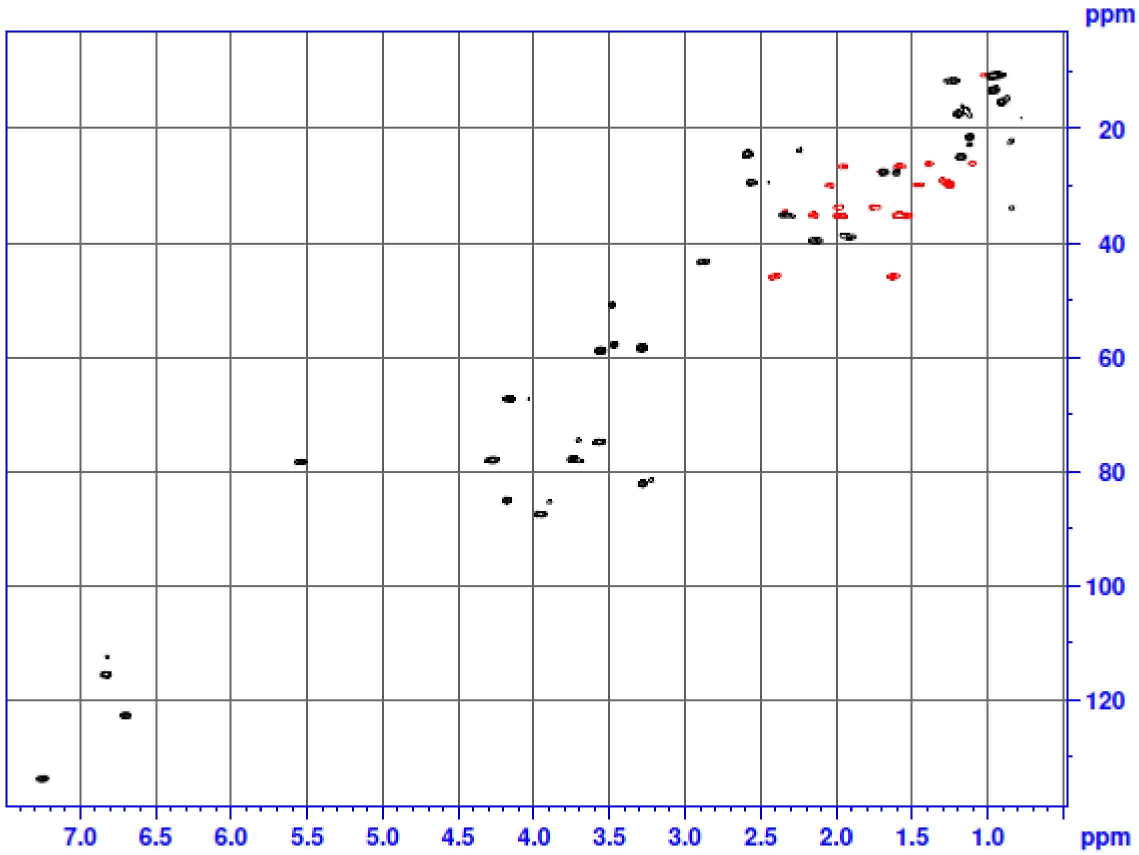

Figure S20. ^{1}H-^{13}C HSQC NMR (400 MHz, $CDCl_3$) spectrum of α-770.

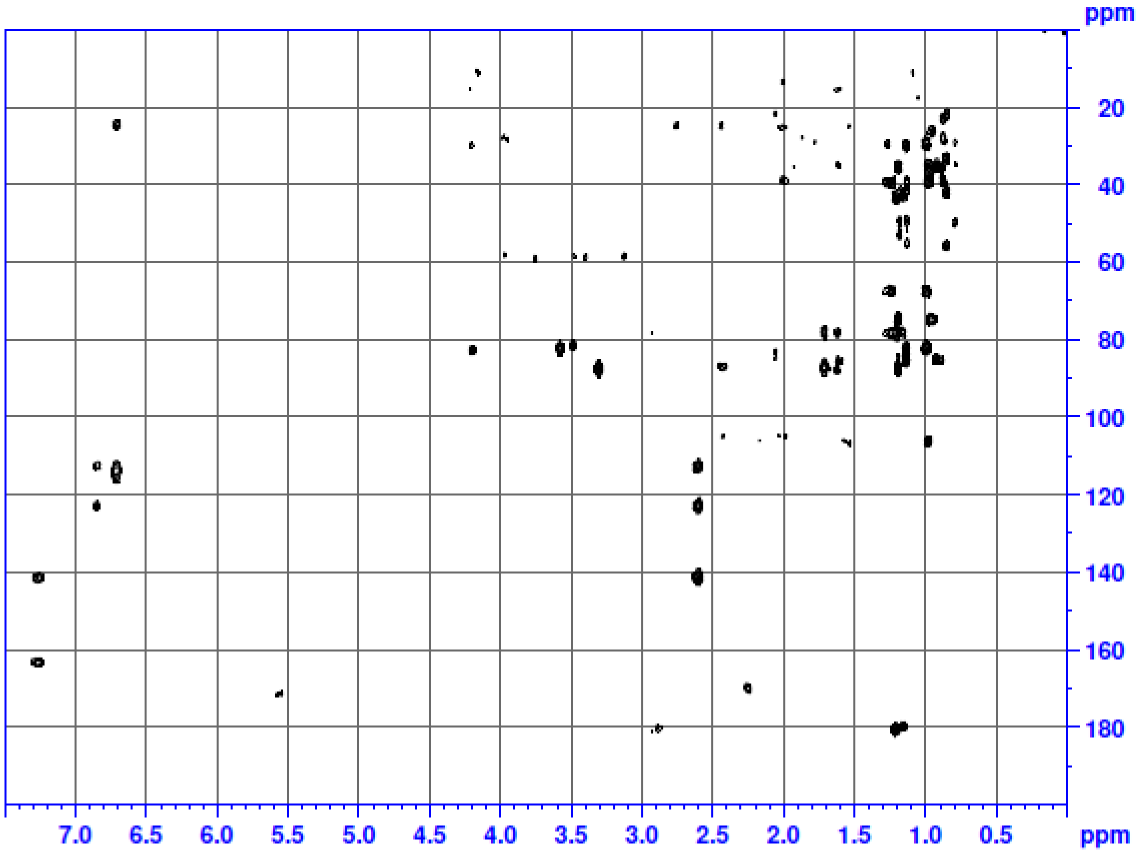

Figure S21. ^{1}H-^{13}C HMBC NMR (400 MHz, $CDCl_3$) spectrum of α-770.

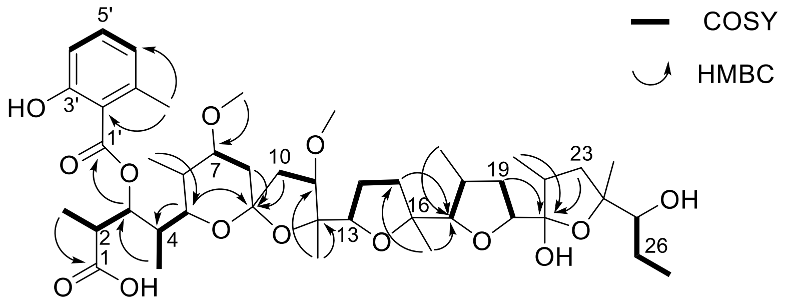

Figure S22. Major COSY and HMBC correlations of α-770.

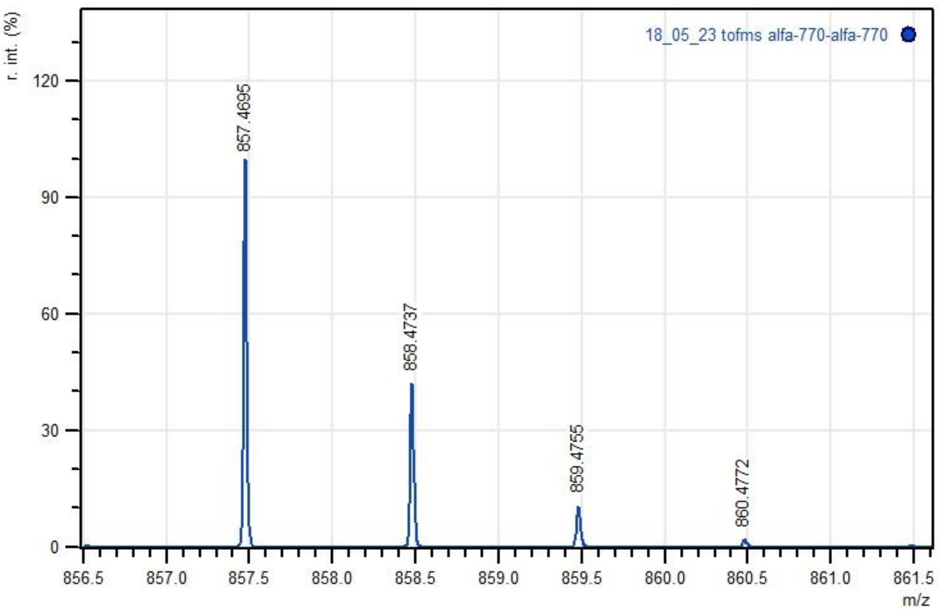

Figure S23. High resolution MS spectrum of α-770.
